# Prise en charge du paludisme en Guyane : quels enjeux dans ce dernier territoire endémique français en 2024 ?

**DOI:** 10.48327/mtsi.v5i1.2025.536

**Published:** 2025-03-20

**Authors:** Laureen DAHURON, Lise MUSSET, Hélène TRÉHARD, Alice SANNA, Aïssata DIA, Yassamine LAZREK, Richard NALDJINAN-KODBAYE, Virginie CÉBRIAN, Luisiane CARVALHO, Yannick ANDRO, Bérengère BONOT, Mathilde BOUTROU, Olivier LESENS, Paul Le TURNIER, Philippe ABBOUD, Brice DAVERTON, Francky MUBENGA, Margot OBERLIS, Jean-Bernard DUCHEMIN, Félix DJOSSOU, Delphine PATAROT, Joseph RWAGITINYWA, Émilie MOSNIER, Maylis DOUINE, Loïc EPELBOIN

**Affiliations:** 1 Unité de maladies infectieuses et tropicales (UMIT), Centre hospitalier de Cayenne (CHC), Guyane, France; 2 Centre national de référence du paludisme, Laboratoire de parasitologie, Centre collaborateur OMS pour la surveillance des résistances aux antipaludiques, Cayenne, France; 3 Aix Marseille Univ, INSERM, IRD, SESSTIM, Sciences économiques et sociales de la santé et traitement de l'information médicale, Aix Marseille Institute of Public Health ISSPAM, F-13385 Marseille, France; 4 Centre d'investigation clinique (CIC), INSERM 1424, Centre hospitalier de Cayenne (CHC), Guyane, France; 5 Direction Interarmées du service de santé (DIASS) en Guyane, Guyane, France; 6 Agence régionale de santé, Guyane, France; 7 Santé publique France, Guyane, France; 8 Pharmacie à usage intérieur, Centre hospitalier de Cayenne (CHC), Guyane, France; 9 Équipe mobile de santé publique en commune (EMSPEC), Centre hospitalier de Cayenne (CHC), Guyane, France; 10 Croix-Rouge française, Cayenne, Guyane, France; 11 Unité Entomologie médicale, Institut Pasteur de la Guyane, Cayenne, France; 12 Laboratoire hospitalo-universitaire de parasitologie mycologie, Centre hospitalier de Cayenne (CHC), Guyane, France; 13 Collectivité territoriale de Guyane (CTG), Guyane, France; 14 Agence nationale de recherches sur le sida, Maladies infectieuses émergentes (ANRS MIE Partner Site), University of Health Sciences, 73 Preah Monivong Blvd Phnom Penh, Cambodia

**Keywords:** Paludisme, *Plasmodium vivax*, Santé publique, Traitement radical, Guyane, Amérique du sud, Malaria, *Plasmodium vivax*, Public health, Radical treatment, French Guiana, South America

## Abstract

La Guyane, dernier territoire endémique du paludisme en France, fait face à une recrudescence épidémique de paludisme depuis fin 2023. Cette épidémie, majoritairement causée par *Plasmodium vivax*, touche principalement des populations éloignées du système de soins. Elle a permis de mettre en lumière les difficultés de délivrance du traitement complet. Celui-ci comprend à la fois un traitement curatif de l'accès par dérivés de l'artémisinine (suite au retrait de la chloroquine du marché) et un traitement éradicateur par primaquine, avec les enjeux d’écarter un déficit en G6PD.

Ce travail a pour objectifs de décrire les problématiques de diagnostic et de prise en charge du paludisme sur ce territoire singulier, de mettre en avant les adaptations réalisées et de proposer des schémas diagnostiques, thérapeutiques et de suivi adaptés aux possibilités d'accès au système de soins dans un but d'homogénéisation des pratiques. Cet article a aussi pour dessein de souligner les stratégies innovantes mises en place en Guyane pour faire face à cette nouvelle épidémie : médiation en santé, équipe mobile « paludisme », tests diagnostiques rapides et traitement immédiat hors les murs *Test and Treat*, développement d'auto-diagnostic et d'auto-traitement. Ces propositions s'intègrent dans une volonté d’élimination du paludisme à court terme sur le territoire français.

## Abstract (en)

French Guiana, the last malaria-endemic region of France, is facing an epidemic resurgence of malaria since the end of 2023. This epidemic, primarily caused by *Plasmodium vivax*, mainly affects populations that are far from the healthcare system. It has highlighted the difficulties of providing a full course of treatment. This includes both curative treatment with artemisinin derivatives (following the withdrawal of chloroquine from the market) and eradicative treatment with primaquine, with the challenge of excluding G6PD deficiency. The aim of this paper is to describe the problems of malaria diagnosis and management in this unique territory, to highlight the adaptations made and to propose diagnostic, therapeutic and follow-up schemes adapted to the possibilities of access to the health system, with a view to homogenizing practices. This article also highlights the innovative strategies implemented in French Guiana to deal with this new epidemic: health mediation, mobile malaria team, rapid diagnostic tests and immediate out-of-hospital treatment Test and Treat, development of self-diagnosis and self-treatment. These proposals are part of a campaign to eliminate malaria in France in the short term.

**Table:** Liste des abréviations

AAC	Autorisation d'accès compassionnel
AAP	Autorisation d'accès précoce
ACT	Associations thérapeutiques à base de dérivés de l'artémisinine
AMM	Autorisation de mise sur le marché
ANSM	Agence nationale de sécurité du médicament
ARS	Agence régionale de santé
ATU	Autorisation temporaire d'utilisation
CDPS	Centre délocalisé de prévention et de soins
CHC	Centre hospitalier de Cayenne
CHK	Centre hospitalier de Kourou
CHOG	Centre hospitalier de l'ouest guyanais
CNR	Centre national de référence
CORRUSS	Centre opérationnel de régulation et de réponses aux urgences sanitaires et sociales
CRF	Croix-Rouge française
CTG	Collectivité territoriale de Guyane
CQ	Chloroquine
DDAS	Direction de la démoustication et des actions sanitaires
DGAS	Direction générale de l'aviation civile
DGS	Direction générale de la santé
EMSPEC	Équipe mobile de santé publique en commune
FAG	Forces armées en Guyane
FMGE	Frottis mince goutte épaisse
G6PD	Glucose-6-phosphate déshydrogénase
HAS	Haute autorité de santé
HCSP	Haut Conseil de la santé publique
IDE	Infirmier.e diplomé.e d’État
IPG	Institut Pasteur de Guyane
IV	Intra-veineux
LAV	Lutte antivectorielle
OMS	Organisation mondiale de la santé
PCR	Réaction de polymérisation en chaîne
PMQ	Primaquine
PUI	Pharmacie à usage intérieur
PUT-RD	Protocole d'utilisation thérapeutique et de recueil des données
SAU	Service d'accueil des urgences
SpF	Santé publique France
SPILF	Société de pathologie infectieuse de langue française
TDR	Test de dépistage rapide
TFN	Tafénoquine
TROD	Test rapide d'orientation diagnostique

## Introduction

**Figure 1:**
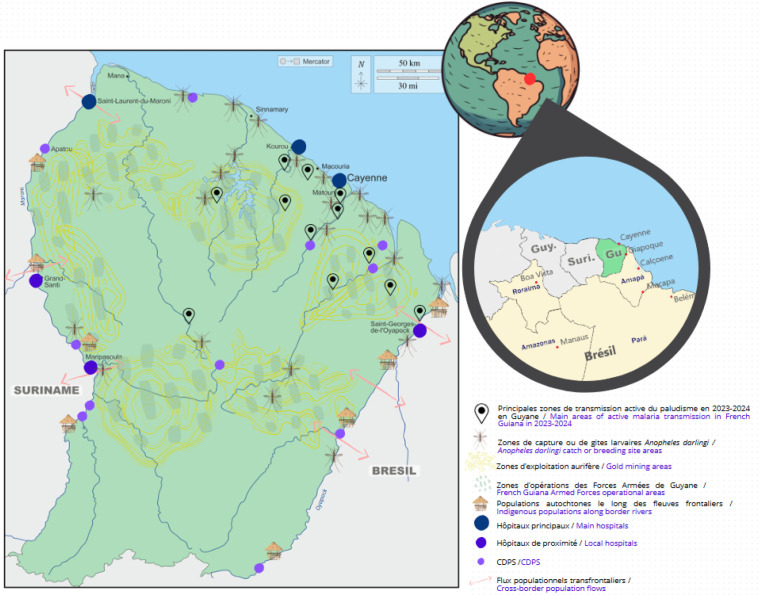
Maillage sanitaire en Guyane et principales zones de transmission du paludisme en 2023-2024

La Guyane, territoire français ultramarin et région ultrapériphérique de l’Union européenne, est située au nord-est de l’Amérique du Sud, à une latitude équatoriale. Elle partage des frontières fluviales au sud-est avec le Brésil (fleuve Oyapock) et au nord-ouest avec le Suriname (fleuve Maroni) (Fig. 1). Ce territoire, appartenant au plateau des Guyanes, reste le dernier bastion de transmission endémique du paludisme en France [29]. En effet, alors que Mayotte ne compte plus de transmission autochtone de paludisme depuis 2020 [51], la circulation active du *Plasmodium vivax* s'est au contraire accélérée en Guyane depuis la fin de l'année 2023 [80].

La situation géographique, économique et sociale de ce territoire complexifie l’élimination du paludisme, pour laquelle la France s'est engagée d'ici 2025, suite aux directives de l’OMS [98, 99]. Son climat équatorial et sa large couverture par la forêt amazonienne – supérieure à 95 % – font que le milieu de vie humain et le milieu naturel sont largement intriqués, y compris pour le littoral urbanisé [30]. Le principal moustique vecteur du paludisme dans la région, *Anopheles darlingi*, est retrouvé en zone péri-forestière, en forêt dégradée ou dans les savanes. Il est responsable de piqûres entre le crépuscule et l'aube, mais de récentes descriptions rapportent des piqûres diurnes avec ce moustique [27, 72]. D'autres espèces d'anophèles potentiellement vectrices sont également décrites comme *An. nuneztovari, An. oswaldoi* ou *An. aquasalis* [12, 28].

La proximité entre les personnes habitant en lisière de forêt et les vecteurs (notamment *An. darlingi)* est constante. Ainsi, entre la fin des années 2010 et le début des années 2020, la transmission du paludisme en Guyane survenait principalement dans deux types de contextes (Fig. 1). D'abord, elle concernait les activités d'orpaillage en zone forestière, où sont également déployés les gendarmes et Forces armées de Guyane (FAG) pour tenter de contrôler ces exploitations aurifères (souvent illégales) [22, 25, 56, 71, 75]. De façon indirecte, cette transmission pouvait aussi s’étendre à des localités abritant des bases arrière logistiques de l'orpaillage. Ensuite, la transmission du paludisme concernait les populations vivant le long des fleuves Oyapock et Maroni, avec d'importants mouvements transfrontaliers et une activité fréquente en zone péri-forestière [33, 62, 64, 65].

La Guyane est également un lieu de diversité ethnique et culturelle du fait de son histoire métissée et des migrations régionales et internationales successives [15, 29, 85]. Ces dernières décennies sont marquées par une explosion démographique (la population totale a doublé en 25 ans, passant de 150 000 habitants en 1997 à environ 300 000 en 2023) [41], accentuant les inégalités sociales. Plus de la moitié de la population vit sous le seuil de pauvreté et 29 % dans la grande pauvreté [42]. La population est majoritairement regroupée sur le littoral et le long des fleuves frontaliers. Environ 60 000 personnes vivent dans les communes de l'intérieur, principalement situées le long des fleuves [41, 85]. L'offre de soins est répartie principalement entre trois hôpitaux sur le littoral : le Centre hospitalier de Cayenne (CHC), le Centre hospitalier de l'ouest guyanais (CHOG à Saint Laurent du Maroni) et le Centre hospitalier de Kourou (CHK). Le maillage sanitaire des communes de l'intérieur est quant à lui constitué de trois hôpitaux de proximité (Maripasoula, Grand-Santi et Saint-Georges de l’Oyapock) et quatorze Centres délocalisés de prévention et de soins (CDPS) reliés au littoral pour la plupart par voie fluviale ou aérienne et coordonnés par le CHC (Fig. 1).

Cet article a pour objectif de mettre en perspective l'application des recommandations françaises sur ce territoire et de décrire les interventions innovantes mises en place dans un contexte géographique, environnemental et social singulier.

## Épidémiologie

### Le paludisme en Guyane

**Figure 2:**
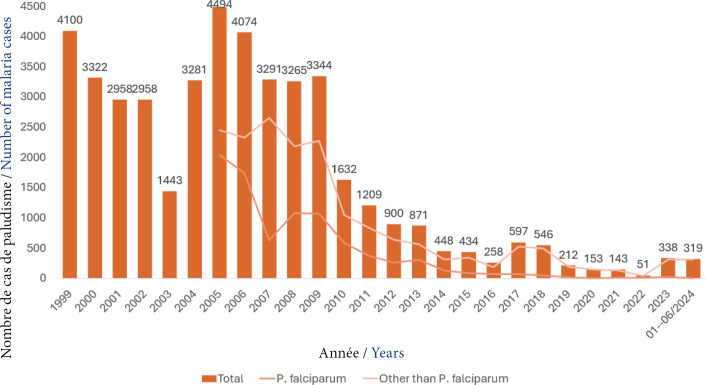
Nombre de cas de paludisme recensés en Guyane, 1999-2024. Sources utilisées : [69, 83, 84, 6, 43]. Données de répartition *P. falciparum* /non *P. falciparum* non disponible avant 2005

La Guyane a enregistré ces dernières décennies une décroissance progressive du nombre de cas de paludisme, passant de plus de 4 000 cas par an dans les années 2000 à 51 cas en 2022 (nadir d'incidence à 0,18 % atteint en 2022) (Fig. 2) [29, 81, 82]. La tendance s'est inversée lors du dernier trimestre 2023, avec une hausse de l'incidence inégalée depuis 2018 (incidence annuelle 2023 : 1,2‰) [79, 80]. *Plasmodium falciparum* était historiquement l'espèce majoritaire jusqu'au milieu des années 2000. Actuellement, *P. vivax* est largement prépondérant (>93 %) [64, 79] (Fig. 2). Quelques cas de *P. malariae* sont décrits de façon plus anecdotique, et généralement transmis en pleine forêt en lien avec un cycle selvatique [94]. Il est important de souligner que le nombre de cas rapportés par les données de surveillance ne reflètent pas complètement les cas réels de paludisme en Guyane, notamment ceux diagnostiqués de chaque côté des fleuves frontières, de même pour les cas déclarés en Europe au retour de Guyane (voyageurs, militaires, etc.) [47, 92]. Les récurrences de *P. vivax* peuvent être expliquées par plusieurs phénomènes : l’échec thérapeutique, la réinfection ou la reviviscence (réactivation des hypnozoïtes hépatiques) [1]. Les reviviscences sont définies comme un accès à *P. vivax* survenant trois semaines à un an après le précédent accès. Elles ont majoritairement lieu dans les trois premiers mois après l'accès initial en Guyane (95 % en 2023), en cohérence avec ce qui est observé dans le continent sud-américain [9, 18, 37, 65]. Les reviviscences concernaient 40 % des cas de l’épidémie survenue entre janvier et juin 2024 (127 accès sur 319 au total) [83].

### Le paludisme en lien avec l'orpaillage

Les orpailleurs clandestins sont une population particulièrement touchée par le paludisme en Guyane. Néanmoins, l’évaluation de la situation épidémiologique sur les sites d'orpaillage reste approximative car les données de surveillance ne reflètent pas la situation épidémiologique réelle. En effet, une grande partie de cette population a recours à l'automédication lorsqu'elle se sent malade ou se rend dans des centres de santé au Suriname ou au Brésil pour des consultations [25]. Depuis la mise en place du projet Malakit (2018-2020) qui consistait en la distribution de kits d'autodiagnostic et d'autotraitement du paludisme aux orpailleurs clandestins, certains d'entre eux se diagnostiquent et se traitent également eux-mêmes sans notification dans le système de surveillance [23]. Plusieurs études transversales (Orpal) descriptives ont été menées en 2015, 2018, 2019 et 2022 sur les points de passage des orpailleurs sur les deux fleuves frontaliers. Les résultats, qui sont un reflet de la situation sur certains sites d'orpaillage, ont montré une prévalence d'une PCR positive de 22,3 % en 2015 contre 5,3 % en 2019 pour la frontière Guyane-Suriname et de 3,9 % en 2018 contre 2,5 % en 2019 pour la frontière Brésil-Guyane. Les données pour 2022 sont en attente. *P. falciparum* était majoritaire (58 %) sur les sites d'orpaillage avant le projet Malakit (2018-2020), tandis que *P. vivax* est désormais prédominant (85 %) [23, 25, 64].

### Les Forces armées de Guyane (FAG), sentinelles du paludisme

Les FAG jouent un véritable rôle de sentinelles de la transmission du paludisme en zone orpaillée. Depuis 2008, des militaires sont impliqués dans des opérations visant à réduire les activités illégales d'extraction d'or en Guyane par la perturbation logistique des exploitations aurifères en forêt primaire via les opérations Anaconda puis Harpie. Pour cela, 400 militaires sont déployés en permanence sur le territoire et se trouvent exposés aux vecteurs infectés, du fait de leur immersion en forêt profonde et de la proximité avec les populations d'orpailleurs, connues pour être un réservoir du paludisme. Ceci impose une stratégie constante de lutte contre le paludisme dans les armées par la protection personnelle antivectorielle, la chimioprophylaxie par doxycy-cline, le diagnostic et le traitement prépositionnés en forêt [21, 25, 73]. Comme décrit ci-après, les FAG ont de nouveau joué un rôle de sentinelle du paludisme dans la bouffée épidémique que connaît la Guyane depuis fin 2023.

### Un cluster guyanais de *P. falciparum* en juin 2023

Bien que *P. falciparum* soit sous-représenté et que les infections de ces dernières années étaient liées à des retours d’Afrique, la Guyane a connu en juin 2023 une alerte avec un foyer de transmission autochtone de *P. falciparum.* Ce foyer est apparu dans une zone péri-urbaine de la commune de Matoury, où les populations d'anophèles sont densément présentes. Les six patients concernés n'avaient pas voyagé et quatre d'entre eux présentaient au moins un facteur clinico-biologique de gravité. Au total, 2/6 patients ont nécessité un transfert en réanimation, aucun décès n'a été rapporté et 2/6 patients ont présenté une rechute 20 et 26 jours après le traitement. Les tests de survie et de chimiosensibilité *in vitro* n'ont pas mis en évidence de résistance parasitaire aux artémisinines et à la luméfantrine. Un des patients présentait des diarrhées au moment du traitement. Seuls les dosages plasmatiques en cours seront en mesure de confirmer la bonne absorption des principes actifs. À noter que ces cas sont survenus dans une zone située à quelques centaines de mètres de l'aéroport international Félix Eboué, mais l'analyse du profil de résistance confirme l'origine amazonienne et la transmission clonale. Grâce à l'aide de médiateurs en santé, une opération de dépistage actif par PCR ultrasensible a pu être proposée à 200 habitants vivant dans un rayon proche des cas. Aucune PCR n'est revenue positive et le foyer s'est éteint avec la prise en soin des patients et une lutte antivectorielle réactive (Données non publiées). Ce scénario souligne le risque de résurgence du paludisme à *P. falciparum*, mettant en évidence l'importance d'un système de surveillance renforcé et d'une prise en charge réactive, à la fois par des équipes mobiles dédiées au paludisme et par des équipes de lutte antivectorielle.

### Une épidémie guyanaise de *P. vivax* depuis octobre 2023

Depuis octobre 2023, la Guyane fait face à une augmentation importante du nombre de cas de paludisme, dont 95 % sont liées à *P. vivax* (Fig. 2). Les 5 % d'accès non liés à *P. vivax* étaient relatifs à des paludismes d'importation. Le premier foyer de l’épidémie de 2023 détecté par le système de surveillance a été mis en évidence parmi les équipes opérationnelles des FAG. Malgré la stratégie de lutte contre le paludisme dans les FAG, 19 % des 378 accès palustres survenus entre octobre 2023 et janvier 2024 concernaient des militaires [80]. Deux clusters majoritaires ont totalisé 40 cas de paludisme à *P. vivax* sur des théâtres d'opérations en forêt profonde avec un taux d'attaque à 89 % (8/9) pour le premier cluster, et 79 % (32/44) pour le second. Cette situation a conduit au rappel et au renforcement des mesures préventives : installation des bivouacs à distance des sites à risque, utilisation de hamacs-moustiquaires imprégnés d'insecticide à longue durée d'action, hygiène corporelle avant la tombée de la nuit pour éviter l'exposition aux piqûres, port de vêtements longs dès la tombée de la nuit, utilisation de répulsifs cutanés, observance stricte de la chimioprophylaxie antipaludique. Dans le reste de la population, des cas de *P. vivax* ont été notifiés dans des zones indemnes depuis plusieurs années sur le littoral et certaines zones péri-urbaines des communes de Kourou, Montsinéry-Tonnégrande et Roura [78, 80]. L’épidémie concomitante massive de dengue a pu également conduire à des retards diagnostiques de paludisme, dus à un système de santé saturé et aux freins à la consultation, les patients n'osant pas consulter [8]. Il s'est ajouté un approvisionnement momentanément insuffisant en traitement par dérivés de l'artémisinine, un arrêt de la commercialisation de la chloroquine et un accès restreint au traitement radical (Encadré 1).

Encadré 1 Problématiques spécifiques de l'accès au traitement radical en Guyane

Depuis février 2022, la HAS a délivré une autorisation d'accès précoce (AAP) qui faisait suite à l’ATU de cohorte de 2020 pour la primaquine qui ne dispose pas d'une AMM. Cette AAP est subordonnée au respect d'un protocole d'utilisation thérapeutique et de recueil des données (PUT-RD), impliquant donc des démarches administratives informatisées pour chaque prescription. Après réception de l'activité en G6PD, le prescripteur réalise la demande d’AAP sur une plateforme web qui nécessite plusieurs informations clinico- biologiques. Chaque demande d’AAP par un clinicien doit être validée par le pharmacien hospitalier et permet la délivrance de la primaquine. Les commandes de médicament impliquent des livraisons aériennes itératives, avec un temps logistique incompressible (démarches d'export, douanes, acheminement…). Les livraisons de primaquine ont par exemple été retardées de plus d'un mois en période de fêtes fin 2023 pour priorisation des livraisons de marchandises alimentaires. Au cours du suivi, le prescripteur doit poursuivre les démarches réglementaires, en remplissant les données clinico-biologiques à J0, J7, J14, et une fiche d'arrêt définitif du traitement. Le défaut de remplissage de ces feuillets par les prescripteurs a conduit à des sanctions par le laboratoire pharmaceutique, diminuant drastiquement les livraisons de primaquine commandées et retardant l'instauration du traitement radical. Sous l'impulsion de l’ARS, des négociations entre l’ANSM et le laboratoire pharmaceutique ont conduit à un assouplissement des procédures de prescription de primaquine : les feuillets de J7 et de J14 ne sont plus à renseigner et les sanctions ont été levées.

D'autres solutions ont été apportées début 2024 pour faciliter la prise en charge globale :

1) Le laboratoire du CHC a validé la mise en place courant avril 2024 de la technique enzymatique de référence du dosage de l'activité en G6PD au sein du laboratoire de Cayenne qui pourrait permettre d'avoir les résultats des dosages de l'activités en G6PD le jour même.
2) Le CNR paludisme de l’Institut Pasteur de la Guyane a réalisé une étude visant à évaluer les performances d'un test quantitatif rapide de l'activité en G6PD (« STANDARD G6PD ») pour la détection des patients déficitaires en comparaison avec la méthode enzymatique de référence. Ce dispositif est simple, rapide et performant. Les valeurs de sensibilité/spécificité du test rapide pour détecter les hommes et les femmes déficitaires sont respectivement de 100 % [89-100] /83 % [73-93] et 100 % [86-100] /90 % [83-97] (Lazrek *et al.* données non publiées). Le déploiement de ces tests capillaires pourrait permettre de débuter le traitement radical dès J0, concomitamment au traitement par chloroquine ou ACT, y compris dans des zones reculées, à l'instar des recommandations brésiliennes [50] (Fig. 6 et Fig. 8). L'usage de cette méthode de diagnostic rapide nécessitera cependant une formation approfondie et suivie de l'ensemble des acteurs du diagnostic, compte tenu des précautions à prendre pour atteindre ce niveau de performance.
3) Sous l'impulsion de l’ARS, une sécurisation du circuit d'acheminement du médicament vers la Guyane s'est organisée pour prioriser les commandes pharmaceutiques. Cette collaboration impliquant la Direction générale de la santé (DGS), le Centre opérationnel de régulation et de réponses aux urgences sanitaires et sociales (CORRUSS) et la Direction générale de l'aviation civile (DGAS) permet de minimiser les retards d'acheminement des médicaments.

Plusieurs leviers sont encore en attente et pourraient avoir des impacts significatifs sur la santé publique.

- L’AMM de la primaquine devrait ainsi être effective dans les mois à venir, ce qui diminuerait drastiquement les temps d'obtention du traitement radical (dès J0), et diminuerait les démarches administratives chronophages pour cliniciens et pharmaciens.
- Le dosage de l'activité en G6PD dès le diagnostic de paludisme aux urgences, pourrait raccourcir la durée avant l'instauration du traitement radical.
- Le raccourcissement de la durée du traitement par primaquine de 14 à 7 jours, mise en place dans de nombreux pays endémiques du paludisme (mais non retenu dans l'avis du HCSP de 2018), pourrait se discuter pour favoriser l'observance [36, 50].
- La disponibilité de la tafenoquine - 8 aminoquino-léine en dose unique - pourrait également représenter un bénéfice majeur dans le parcours de soins en minimisant le temps de suivi et limitant le nombre de perdus de vue avant traitement radical [9, 10, 16]. Sa mise sur le marché en France ne semble pour l'instant pas à l'ordre du jour.Enfin, dans les zones d'endémies palustres à *P. vivax*, un dosage systématique du G6PD pourrait être réalisé à l'occasion d'un bilan chez toute personne habitant dans les zones géographiques à risque et notifié dans le dossier médical afin de faciliter la mise en place d'un traitement curatif en cas d'accès palustre (Fig. 8).

### Contexte régional depuis 2023 : évolution récente sur le plateau des Guyanes

L’évolution épidémiologique observée en Guyane entre 2023 et le premier trimestre de 2024 doit être interprétée dans le contexte régional et continental. En 2023, une importante épidémie s'est déclarée dans l’État brésilien frontalier de l’Amapá qui a particulièrement touché les municipalités de Calçoene (907 cas en 2022 vs 2 637 en 2023) et Oiapoque, ville frontière avec la Guyane (454 cas en 2022 vs 892 cas en 2023) [58]. Cette épidémie avait son épicentre dans le district du Lourenço (Calçoene), secteur historique d'extraction légale et informelle d'or [50] (Fig. 1). Elle s'est caractérisée par une prépondérance de *P. vivax* qui représentait 95 % des cas notifiés [93]. À l'inverse, le Suriname a connu une année stable et une absence de transmission autochtone sur le territoire depuis 2021 [67].

À l’échelle amazonienne, voire continentale, deux autres phénomènes peuvent avoir influencé la dynamique épidémiologique guyanaise. D'une part, on a constaté des mouvements populationnels difficiles à quantifier, au départ des aires d'orpaillage illégal de la réserve amérindienne Yanomami située dans l’État brésilien du Roraima, ayant fait l'objet d'importantes opérations militaires en début d'année 2023 dans le cadre de la politique du Président Lula de renforcer la protection des populations autochtones [49, 48]. Ces mouvements auraient contribué à disperser une population fortement touchée par *P. vivax* vers d'autres bassins d'orpaillage en Amazonie, parmi lesquels l’Amapá et la Guyane [20, 54]. D'autre part, le phénomène climatique El Niño a touché l'ensemble du continent américain, se traduisant en Amazonie par une année exceptionnellement sèche et chaude [55, 98], propice à la création de retenues d'eau, créant un environnement favorable à la multiplication vectorielle. On peut également évoquer un impact de l'arrêt des études interventionnelles sur les sites d'orpaillage tel que l'arrêt du projet Malakit en 2020 (distribution aux orpailleurs illégaux de kits d'autodiagnostic et d'autotraitement du paludisme [cf infra]) [23]. Ainsi, l'origine de l’épidémie de *P. vivax* actuelle en Guyane est probablement multifactorielle, et mériterait d’être étudiée plus avant.

### Gérer l'élimination et le risque de réintroduction

Avec la décroissance des cas de paludisme entre 2000 et 2022, la Guyane est entrée dans la phase d’élimination du paludisme (c'est-à-dire aucune transmission palustre autochtone pendant trois années consécutives). Cet objectif d’élimination vise, à la différence d'un programme de contrôle, à interrompre la transmission puis à empêcher sa réintroduction [59, 96]. La route vers la certification OMS inclut trois grandes phases : 1) un dépistage et un traitement réactif des cas, des individus co-exposés et de leur environnement, 2) la détection et la réponse ciblée des populations considérées comme réservoir, et 3) la prévention du risque d'introduction ou de réintroduction par le dépistage de personnes provenant de zones endémiques aux points d'entrée du territoire [89, 96, 99]. Les zones frontalières de la Guyane restent très vulnérables au paludisme, du fait notamment de l'importante mobilité des populations et du contexte endémique régional global perpétuant les éventuelles transmissions [2, 17, 26, 60, 62, 76, 89]. Du fait des phénomènes épidémiques 2023-2024, l'objectif OMS d’élimination du paludisme pour 2025 en Guyane est compromis.

## Clinique

Les infections à *P. vivax* sont généralement décrites comme moins sévères que *P. falciparum.* Cependant, la fréquence des cas graves diffère selon les zones géographiques et les sensibilités aux antipaludiques, allant de 0,6 % à 21 % dans la littérature [36, 45, 63, 70, 88]. En Guyane, la proportion des infections sévères à *P. vivax* était estimée à 4 % entre 2018 et 2020 [18]. L’épidémie actuelle semble présenter un profil légèrement différent. En effet, parmi les 371 cas de paludisme à *P. vivax* identifiés entre octobre 2023 et janvier 2024, 17 % ont été hospitalisés (62/371) dans un des trois principaux hôpitaux de Guyane. Parmi les 34 patients hospitalisés au CHC, seize patients (47 %) ont présenté au moins un facteur de gravité selon les critères OMS. On retrouvait en majorité des ictères (7), des détresses respiratoires (6) et des défaillances circulatoires (4). Parmi les 34 patients hospitalisés, 5 ont nécessité un transfert en réanimation (15 %), 8 patients ont reçu un traitement par artésunate IV, mais aucun décès n'a été constaté [19]. Des compléments d’étude seront nécessaires pour mieux décrire ces éléments de gravité inattendus sur l'ensemble du territoire.

## Diagnostic parasitologique

### Méthodes diagnostiques

Tout patient présentant une fièvre sans point d'appel en Guyane doit systématiquement bénéficier d'une recherche de paludisme. Le diagnostic est réalisé au laboratoire et le résultat doit être rendu dans les deux heures suivant la réception de l’échantillon. Celui-ci se base sur les recommandations françaises associant une technique sensible (goutte épaisse ou biologie moléculaire) à un frottis mince sanguin, ou à défaut, un test de diagnostic rapide (TDR) associé à un frottis mince [86]. Le TDR principalement utilisé en Guyane est le Sd Bioline Antigen Pf/Pan (HRP2/pLDH). Sa sensibilité pour diagnostiquer *P. falciparum* est très bonne d'autant plus qu'aucune délétion HRP2 n'a été retrouvée à l'heure actuelle sur le plateau des Guyanes [10]. La sensibilité pour diagnostiquer *P. vivax* est décrite par l’OMS à 91,7 % nécessitant de répéter la recherche de paludisme sur un nouveau prélèvement en cas de forte suspicion clinique [87, 97]. Dans les hôpitaux de proximité ou dans les CDPS, le TDR réalisé sur place permet d'entreprendre un traitement en cas de positivité sans attendre la microscopie. Chaque cas positif doit secondairement faire l'objet d'un envoi au laboratoire du CHC pour confirmation microscopique et détermination de la parasitémie, puis transmission au CNR paludisme.

### Utilisation hors-les-murs du TDR paludisme

Dans les zones éloignées des structures de soins, la confirmation diagnostique se complexifie. En effet, les TDR rentrent dans le cadre de la biologie délocalisée et devraient donc être confirmés par un biologiste. Cependant, l'arrêté dérogatoire propre au territoire guyanais du 1^er^ décembre 2016 (révisé en avril 2024), a permis de simplifier la prise en charge en donnant au TDR paludisme le statut de test rapide d'orientation diagnostique (TROD). Aussi, après une formation spécifique et habilitation, les IDE et le personnel relevant de structures de soins ou de prévention, peuvent réaliser les examens de détection antigénique du paludisme dans les lieux éloignés de tout laboratoire de biologie médicale de Guyane [57].

### Dépistage actif par PCR ultrasensible

**Figure 3:**
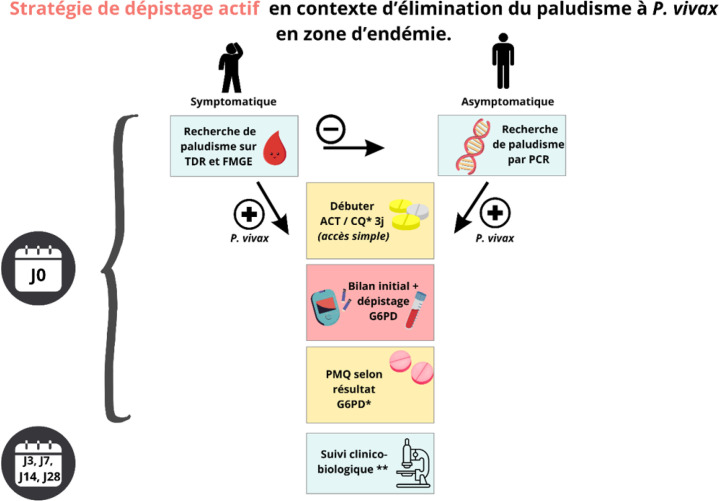
Stratégie de dépistage actif mise en œuvre en Guyane TDR : Test de dépistage rapide; FMGE : Frottis mince goutte épaisse; PCR : Réaction de polymérisation en chaîne; ACT : Thérapies combinées à base d'artémisinine; CQ : Chloroquine; G6PD : Glucose-6-phosphate déshydrogénase; PMQ : Primaquine; J : Jour * en l'absence de contre-indication ** adapté au contexte et à l'éloignement géographique

La PCR ultrasensible est quant à elle utilisée dans le cadre du dépistage actif (Reactive Test and Treat) chez des patients asymptomatiques dans des situations épidémiques particulières. Il s'agit de réaliser une PCR à toute personne résidant dans une zone identifiée « à risque » (par une évaluation collégiale) et de procéder au traitement des individus positifs (Fig. 3). Ces dépistages actifs sont coordonnés par l’ARS, et la PCR est réalisée par le CNR à l’IPG. Le dernier en date a eu lieu en février-mars 2024 dans le village amérindien de Favard (village de la commune de Roura). Sur 138 habitants dépistés, 11 patients étaient symptomatiques et positifs en TDR (8 %). Quatre patients asymptomatiques ont pu être dépistés par PCR motivant la mise en place d'un traitement. L'objectif final est de traiter immédiatement l'ensemble des cas diagnostiqués au décours des dépistages actifs pour limiter au plus vite la transmission du paludisme.

## Traitement

### Traitement de l'accès à *P. falciparum*

**Figure 4:**
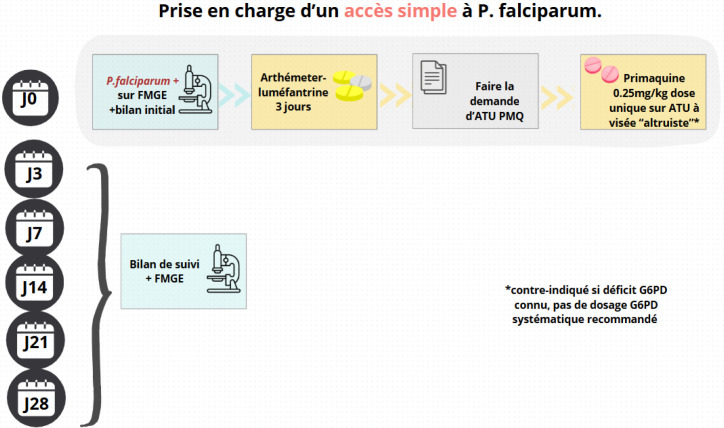
Propositions de prise en charge d'un accès simple à P *falciparum* en zone d'endémie FMGE : Frottis mince goutte épaisse; PMQ : Primaquine; J : Jour

**Figure 5:**
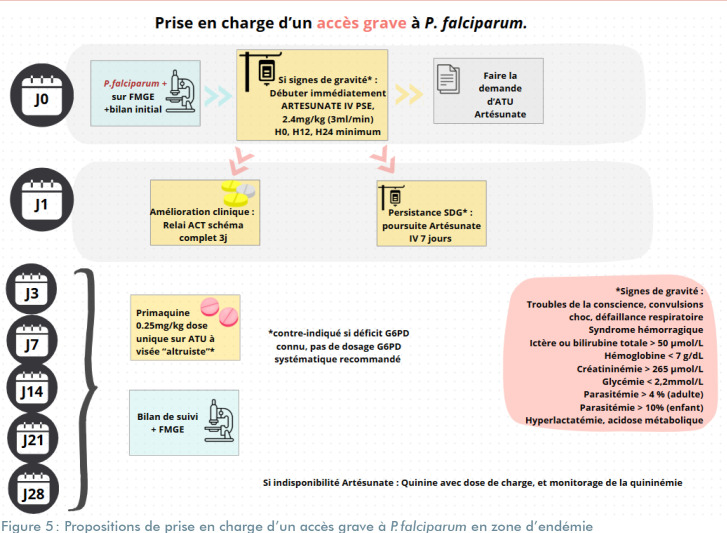
Propositions de prise en charge d'un accès grave à *P. falciparum* en zone d'endémie FMGE : Frottis mince goutte épaisse; ACT : Thérapies combinées à base d'artémisinine; G6PD : Glucose-6-phosphate déshydrogénase; ATU : Autorisation temporaire d'utilisation; PMQ : Primaquine; SDG : Signes de gravité; PSE : Pousse-seringue électrique; J : Jour

La prise en charge des accès palustres à *P. falciparum* s'appuie sur les recommandations nationales et internationales, avec un traitement par artéméther-luméfantrine (20 mg/120 mg, 4 comprimés à H0, H8, H24, H36, H48, H60 chez l'adulte) en l'absence de critères de sévérité, ou par artésunate IV si des critères de sévérité sont présents [30] (Fig. 4 et Fig. 5). Comme recommandé par le HCSP face à l'objectif d’élimination du paludisme, les patients atteints de *P. falciparum* devraient recevoir une dose unique de primaquine (0,25 mg/kg) à visée « altruiste » pour son action anti-gamétocytaire afin de réduire la transmission du parasite aux anophèles vecteurs [5, 38].

L'association arténimol-pipéraquine largement utilisée dans l’Hexagone, a été déployée fin des années 2010 dans les FAG. Son utilisation a été suspendue suite à l'identification de nombreux échecs thérapeutiques liés à la proportion non négligeable de *P. falciparum* résistants à la pipé-raquine sur le plateau des Guyanes [32]. Bien que très peu utilisée dans le système de soins, la résistance à la pipéraquine a pu être sélectionnée *via* l'utilisation massive en automédication par les orpailleurs du plateau des Guyanes depuis le début des années 2000 [24].

### Traitement de l'accès à *P. vivax*

**Figure 6:**
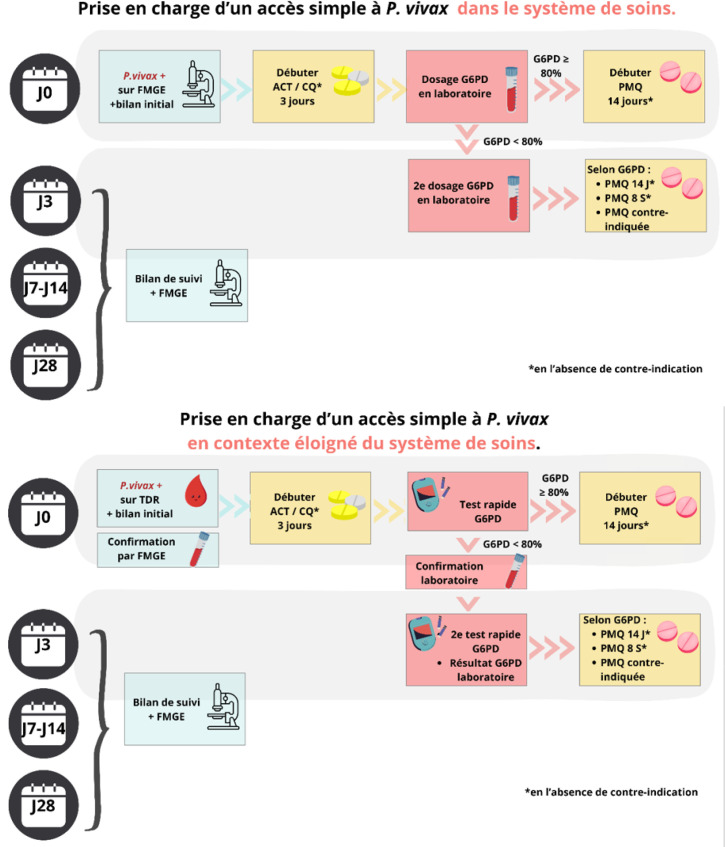
Propositions de prise en charge d'un accès simple à P. *vivax* en zone d'endémie, en fonction de l'accès au système de soin TDR : Test de dépistage rapide; FMGE : Frottis mince goutte épaisse; ACT : Thérapies combinées à base d'artémisinine; CQ : Chloroquine; G6PD : Glucose-6-phosphate déshydrogénase; PMQ : Primaquine; J : Jour

**Figure 7:**
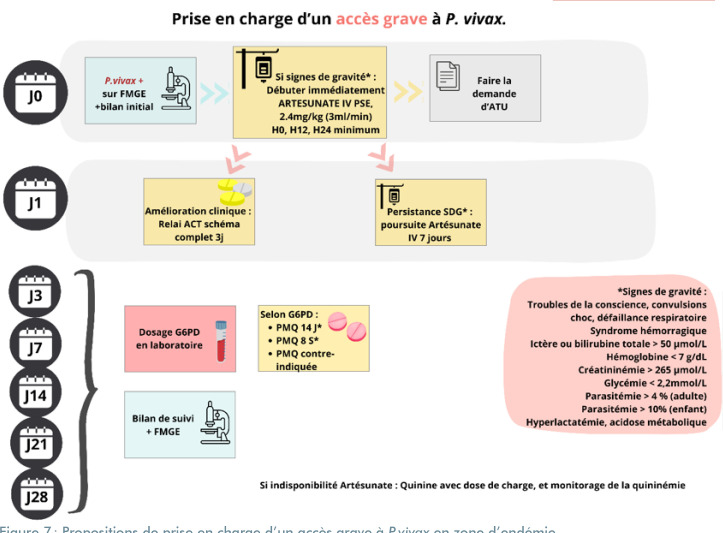
Propositions de prise en charge d'un accès grave à *P.vivax* en zone d'endémie FMGE : Frottis mince goutte épaisse; ACT : Thérapies combinées à base d'artémisinine; G6PD : Glucose-6-phosphate déshydrogénase; ATU : Autorisation temporaire d'utilisation; PMQ : Primaquine; SDG : Signes de gravité; PSE : Pousse-seringue électrique; J : Jour

Les accès palustres à *P. vivax* étaient jusqu’à récemment traités par chloroquine à la dose de 25 mg/kg sur 3 jours (J1 : 10 mg/kg/j - J2 : 10 mg/kg/j - J3 : 5 mg/kg/j). La fin de sa commercialisation en 2022 a nécessité de modifier les protocoles. Ainsi, l'artéméther-luméfantrine (20 mg/120 mg) est actuellement utilisé à la même posologie que pour les accès à *P.falciparum* [29, 30] (Fig. 6 et Fig. 7). Ce changement de paradigme parait avoir des conséquences en termes d'observance et d’échecs thérapeutiques. Pour rappel, un échec thérapeutique est actuellement défini par Santé publique France comme un deuxième accès survenant dans les trois semaines suivant l'accès précédent [79].

En effet, parmi 59 militaires traités par ACT, 61 % (36/59) ont subi un 2^e^ accès – majoritairement dans le premier mois – alors qu'ils ne se seraient pas réexposés sur la période (19 % d’échecs thérapeutiques, 81 % de reviviscences). Les rechutes surviennent précocement : 27 jours en médiane chez les patients suivis en maladies infectieuses, alors qu'elles survenaient 63 jours en médiane dans la même population traitée par chloroquine, en 2018-2020 [18]. À noter également plusieurs reviviscences après un traitement bien conduit chez des patients initialement traités par ACT (Données non publiées).

Pour faire face à la crise sanitaire actuelle et suite à l'arrêt de la commercialisation de la chloroquine, l’ARS de Guyane a obtenu sa mise à disposition sous le statut d'autorisation d'accès compassionnel (AAC). La chloroquine est en effet une molécule majeure du fait de son efficacité sur les accès à *P. vivax*, de la diminution potentielle du risque d’échecs thérapeutiques et de reviviscence et de son effet synergique avec la primaquine [3, 16, 35, 46, 74]. Elle est ainsi disponible à la pharmacie hospitalière après remplissage des feuillets réglementaires. Le retour d'une AMM pour la chloroquine est urgent afin de pouvoir disposer de ce traitement sur l'ensemble du territoire (y compris en médecine de ville et dans les zones isolées) et de pouvoir l'administrer sans délai.

### Traitement radical

*Plasmodium vivax* produit des hypnozoïtes hépatiques pouvant être responsables de reviviscences quelques semaines à quelques mois après le traitement par schizonticide. Pour les éradiquer efficacement et prévenir les reviviscences, on utilise un traitement radical par une 8-aminoquinoléine, dont seule la primaquine est disponible en France. La primaquine peut être responsable d'anémie hémolytique en cas de déficit sévère en glucose-6-phosphate-déshydrogénase (G6PD). Un dosage de l'activité en G6PD est donc recommandé par l’OMS et requis par l’Agence nationale de sécurité du médicament (ANSM) avant toute prescription. Le Haut Conseil de la santé publique (HCSP) préconise de doser l'activité G6PD à J14 du début de l'accès palustre, pour s’éloigner de l'hémolyse initiale qui pourrait surestimer le dosage en G6PD. Cependant, le suivi du traitement radical en Guyane fait face à un nombre important de perdus de vue, conduisant à des reviviscences répétées, pouvant perpétuer les chaînes de transmission. Pour illustrer, sur 113 accès palustres étudiés entre 2018 et 2020 au CHC, le temps médian entre le traitement par chloroquine ou ACT et celui par primaquine était de 28 jours. Sur les 113 accès palustres, 30 patients (27 %) ont été perdus de vue avant traitement par primaquine et 40 patients (3 5%) ont subi au moins une reviviscence [18]. C'est dans ce cadre qu'a été étudiée la possibilité de réaliser des dosages précoces en G6PD (dès le diagnostic de paludisme). Cette étude rétrospective ne retrouvait pas de variations dans l'activité de la G6PD entre l'initiation du traitement et J28 : aucun patient classé à la phase initiale comme non déficitaire en G6PD n’était retrouvé déficitaire à J14. Au contraire, elle mettait en évidence des cas de faux positif : patients faussement déficitaires en G6PD à la phase initiale, finalement normalisés au cours du suivi. Ceci suggérerait la possibilité de doser précocement l'activité en G6PD au cours d'un accès à *P. vivax* et de recontrôler ce dosage en cas de déficit initial [18].

**Figure 8:**
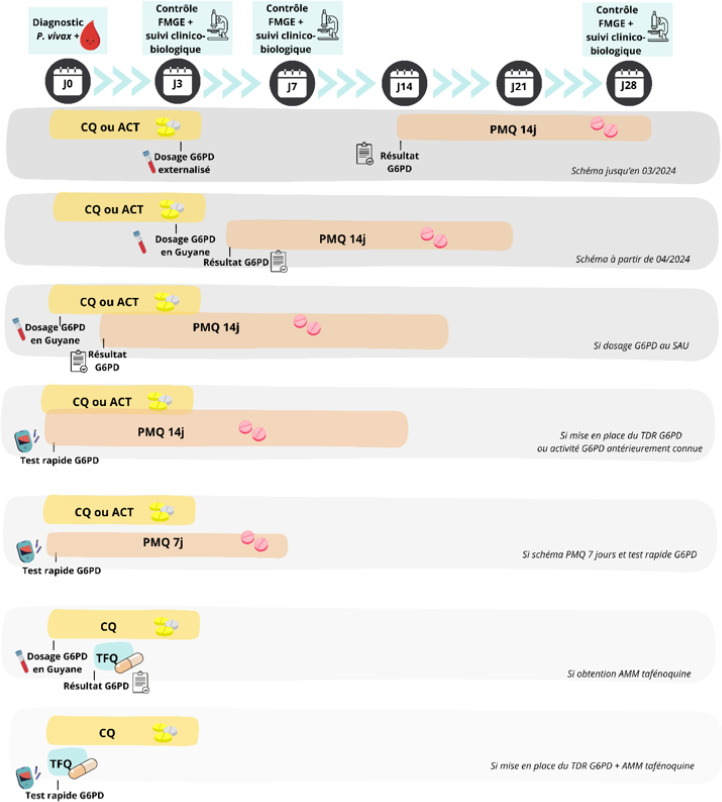
Propositions de différents schémas diagnostiques et thérapeutiques de prise en charge des accès à P. *vivax* en Guyane FMGE : Frottis mince goutte épaisse; ACT : Associations thérapeutiques à base de dérivés d'artémisinine; CQ : Chloroquine; G6PD : Glucose-6-phosphate déshydrogénase; PMQ : Primaquine; TFN : Tafénoquine; AMM : Autorisation de mise sur le marché; TDR : Test de dépistage rapide; SAU : Service d'accueil des urgences

**Figure 9:**
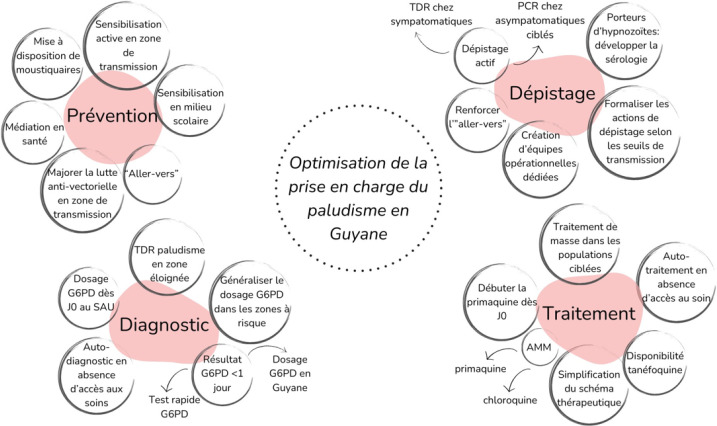
Leviers d'optimisation de la prise en charge du paludisme pour atteindre l'élimination, face aux enjeux du territoire

**Figure 10:**
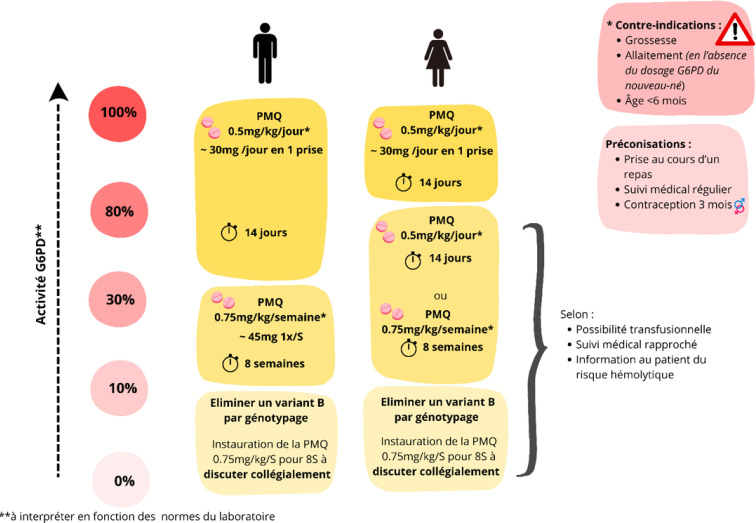
Traitement radical d'un accès simple à *P.vivax* chez l'adulte en fonction de l'activité G6PD selon les recommandations du HCSP 2018 [4, 39, 99] G6PD : Glucose-6-phosphate déshydrogénase; PMQ : Primaquine; U : Unité; Hb : Hémoglobine ** Exemple : si norme G6PD du laboratoire = 10 U/g Hb : G6PD 10 U/g Hb = 100 % d'activité G6PD; G6PD 8 U/g Hb = 80 % d'activité G6PD; G6PD 3 U/g Hb = 30 % d'activité G6PD; G6PD 1 U/g Hb = 10 % d'activité G6PD.

Au début de l’épidémie palustre de 2023 en Guyane, des obligations administratives et des difficultés logistiques (Encadré 1) se sont ajoutées aux difficultés de suivi déjà présentes, retardant considérablement la mise en place du traitement radical. C'est dans ce contexte et suite aux récentes données rassurantes de la littérature [13, 18, 52] que le dosage précoce de l'activité en G6PD a été mis en place, sous couvert d'une information détaillée au patient et d'un suivi clinico-biologique rapproché (Fig. 6). Plusieurs autres leviers d'optimisation diagnostique et thérapeutique sont encore en attente et pourraient avoir des impacts significatifs sur la santé publique (Encadré 1, Fig. 8, Fig. 9). En Guyane, comme en Amérique du Sud, la prévalence du déficit en G6PD est faible, <2 % [11, 18]. Le dosage de G6PD est exprimé en unités d'enzyme par gramme d'hémoglobine (U/g Hb). L'interprétation de l'activité G6PD se fait quant à elle en pourcentage. Le clinicien doit donc déduire l'activité en pourcentage, en fonction de la norme donnée par chaque laboratoire. Par exemple, selon les normes actuellement utilisées par le laboratoire Biomnis qui traite les prélèvements en Guyane, l'activité à 100 % de la G6PD correspond à la valeur seuil de normalité donnée par le laboratoire de 10 U/g Hb (donc 8 U/g Hb – 80 %, 3 U/g Hb – 30 %, 1 U/g Hb – 10 %). Le schéma posologique de primaquine selon l'activité G6PD en fonction des recommandations du HCSP est décrit dans la Figure 10.

### Prise en charge sur le littoral

Le diagnostic et l'initiation du traitement initial (ACT ou CQ) dans le cadre d'accès palustres à *P. vivax* sur le littoral sont souvent réalisés au service d'accueil des urgences ou en médecine de ville. Les patients sont adressés en consultation de maladies infectieuses à J3 pour la suite de la prise en charge : dosage de l'activité en G6PD, instauration de la primaquine et suivi régulier jusqu'en fin de traitement – J14 de primaquine – (Fig. 8). L'organisation actuelle retarde le dosage en G6PD à J3 et donc le traitement radical. Un protocole avec dosage de la G6PD dès les urgences est à discuter en cas d'accès à *P. vivax* pour optimiser le traitement radical.

### Prise en charge dans les communes isolées

La prise en charge du paludisme dans les communes de l'intérieur fait face à l'isolement de leurs populations. Les patients peuvent mettre plusieurs heures ou jours de pirogue ou de quad pour rejoindre le centre de santé, complexifiant le suivi médical [14, 85]. À cela s'ajoutent les difficultés d'accès au traitement radical (Encadré 1), les difficultés à joindre les patients une fois le traitement réceptionné (utilisation de numéros de téléphone étrangers, réseau téléphonique incertain…) et les enjeux logistiques. Les pirogues ou les avions reliant les CDPS au littoral ont des rotations parfois hebdomadaires. Leur régularité est assujettie aux conditions climatiques (niveaux d'eaux fluviales, conditions de vol) ou organisationnelles (faillite récente du transporteur aérien intra-Guyane, grèves aériennes, etc.). Ainsi, malgré l'intervention des médiateurs en santé chargés d'aller vers les patients, l'ensemble de ces difficultés conduisent à de multiples pertes de vues et à une très faible couverture par primaquine [34]. Il reste possible que ces pertes de vue soient finalement traitées au Brésil ou au Suriname du fait d'importants mouvements populationnels transfrontaliers, mais ceci reste incertain et non quantifiable. Les CDPS souffrent également d'un manque de ressources humaines et d'un important *turn over* des équipes médicales et paramédicales, ce qui complique la continuité du parcours de soins [15]. La complexité réside aussi dans la formation de ces professionnels de santé – non-infectiologues, souvent issus de l’Hexagone – qui doivent comprendre dans un temps record les spécificités des communautés qu'ils rencontrent et les particularités des différentes épidémies rencontrées.

### Place de la chimioprophylaxie

Bien qu'elle soit encore mentionnée par le HCSP et les recommandations de la SPILF, la chimioprophylaxie contre le paludisme lors d'un voyage en Amérique du Sud est limitée à quelques exceptions [86]. En effet, le bénéfice-risque de la chimioprophylaxie en zone de faible risque de transmission à *P.falciparum* est le plus souvent défavorable. Les situations qui pourraient la justifier comprennent les déplacements dans des zones à risque, les situations d'isolement en forêt et les conditions de séjour particulières (par exemple, les militaires intervenant en forêt). Ces informations sont régulièrement mises à jour sur le site de l’ARS Guyane qui publie la carte du risque du paludisme en Guyane coordonnée par SpF [7, 29].

## Interventions à l'échelle individuelle et collective

Dans un contexte d’élimination, la simple gestion des cas de paludisme identifiés par la surveillance passive (personnes recherchant du soin dans le contexte de symptômes) n'est pas suffisante. La mise en place d'interventions proactives dans les poches récentes de transmission est nécessaire pour l'interruption de la transmission. Dans ce paragraphe, nous décrivons des expériences guyanaises ainsi que quelques perspectives d'actions potentiellement pertinentes dans l'avenir pour la lutte contre le paludisme dans la région (Fig. 8).

### Médiation en santé et « aller-vers »

Les équipes mobiles de santé publique en commune (EMSPEC) du CHC ont été créées en 2019 suite à l'intérêt observé lors de l’étude PALUSTOP (cf. infra) et de l’épidémie de paludisme à Saint-Georges de l’Oyapock en 2017. Ces équipes ont pour but de répondre aux besoins de santé publique des populations des communes de l'intérieur et sont rattachées aux hôpitaux de proximité et aux CDPS [34, 85]. Chaque équipe est constituée de binômes médiateur(s)-infirmier(s) et elle est coordonnée par un trinôme médecin(s)-infirmier(s). La médiation en santé est placée au cœur des actions de promotion et de prévention grâce aux médiateurs issus des communautés qui adaptent les actions aux besoins et aux cultures locaux. L’EMSPEC a un rôle pivot dans la lutte contre le paludisme grâce à sa démarche d’« aller-vers » et d'interventions « hors les murs ». Dès lors qu'un patient présente un accès palustre, les équipes le rencontrent à domicile, réalisent l'enquête autour du cas, insistent sur l'importance du suivi, distribuent des moustiquaires imprégnées d'insecticide (fournies par l’ARS) quand elles sont disponibles, et accompagnent le patient tout au long du parcours de soins. Elles sensibilisent aussi de manière globale sur le paludisme et la lutte antivectorielle *via* des stands ou des maraudes et participent à la réduction des inégalités sociales et territoriales sur des sujets variés. Face à la nouvelle répartition géographique des cas – et sous l'impulsion de l’ARS –, quatre binômes infirmier-médiateur devraient renforcer cette approche au sein de la Croix-Rouge française et du CHC au niveau du secteur littoral et dans les populations d'orpailleurs : sensibilisation, dépistage par TDR *in situ*, orientation vers le parcours de soin, distribution de moustiquaires, etc.

### Dépistage et traitement des personnes considérées à risque dans les populations ciblées

Face à la situation épidémiologique à Saint-Georges de l’Oyapock en 2017, il a été mis en place un dépistage de masse en population générale lors de l’étude PALUSTOP. Ce projet innovant a permis d’évaluer une stratégie de dépistage et de traitement en étudiant le portage du paludisme (symptomatique et asymptomatique) par PCR chez une cohorte de plus de 1 200 personnes suivies pendant plus d'un an [62]. Cette étude a permis d'analyser l'intérêt des différents outils diagnostic, sérologie, TDR et PCR. Alors que les TDR manquaient de sensibilité pour détecter *P. vivax* lors de dépistage actif (9,9 % des 91 cas positifs en PCR détectés par les TDR), leur association à de nouveaux outils (PCR ou sérologie) semblait pertinente dans une stratégie d’élimination [60, 91]. En effet, si le TDR est suffisamment sensible pour détecter les patients symptomatiques, sa sensibilité est diminuée dans les formes pauci ou asymptomatiques, notamment en cas de faible parasitémie [90]. Cette stratégie de dépistage de masse et de traitement pourrait être amenée à être répétée dans le contexte épidémique actuel. Cette étude a permis de spatialiser le risque de paludisme à la périphérie de ce village transfrontalier [62] et d'identifier certains profils à risque (jeunes Amérindiens) ou facteurs de risques (activités d'exploitation forestière, voyages en zone amérindienne brésilienne) [62, 89]. Ces résultats ont souligné l'importance de coordonner les efforts de lutte transfrontalière et ont motivé la création d'un système de surveillance épidémiologique entre la Guyane et le Brésil [76]. Une analyse ethno-pharmacologique et une enquête sur les connaissances et les pratiques ont révélé d'importantes disparités entre les groupes ethniques, ce qui implique de personnaliser les outils de prévention et de prise en soin, en menant des actions participatives et en impliquant des médiateurs en santé communautaires [33, 61, 66]. Le dépistage actif par PCR autour des cas fait partie des stratégies d'intervention déployées par l’ARS suite à des signaux épidémiologiques ou alertes sanitaires. Ce modèle interventionnel, appuyé par l’OMS dans ses grandes lignes, a été mis en œuvre pour la première fois – en dehors du contexte de recherche – en 2023 en Guyane.

Il nécessite une collaboration étroite entre l’ARS, l’équipe mobile paludisme de la CRF (créée en avril 2024 et constituée d'une coordinatrice de projets, d'un infirmier(e) et de médiateurs), les médecins du CHC ou CHK, le laboratoire du CHC, le CNR paludisme de l’IPG et les médiateurs en santé (EMSPEC ou bénévoles de l'association DAAC). Ce modèle est actuellement déployé de façon hebdomadaire sur un des sites de repli d'une zone orpaillée proche de Kourou, lieu de transit important de populations reculées et fortement impactées par le paludisme. En pratique, les personnes symptomatiques et positives en TDR sont traitées immédiatement par ACT et primaquine après avoir bénéficié d'un TDR G6PD pour éliminer un éventuel déficit. Le diagnostic est confirmé a posteriori par frottis et goutte épaisse. Le dépistage actif par PCR est proposé aux personnes négatives en TDR et vivant dans un périmètre proche des cas positifs ou aux personnes ayant eu un lien avec l'orpaillage dans les 12 derniers mois; le traitement est organisé en cas de positivité. Un suivi clinique et biologique hebdomadaire est réalisé sur site pour tous les patients sous primaquine (Fig. 3). Cette intervention a permis de dépister de nombreux cas de paludisme et doit être évaluée pour permettre d'organiser sa pérennité et d'affiner la balance coût-bénéfice [31, 96].

Néanmoins, si les phases aiguës de l'accès palustre sont facilement dépistées, aucun test biologique ne permet de détecter le portage de formes dormantes (hypnozoïtes). Ceci constitue le principal écueil à l’élimination du *P. vivax*. De fait, des personnes récemment infectées et à risque de reviviscences peuvent échapper aux dépistages de masse par TDR, PCR ou microscopie. Des stratégies de traitement de masse (ciblées ou non) pourraient sembler pertinentes en vue de l’élimination, mais elles sont à contrebalancer avec les contre-indications du traitement radical [40, 44, 68]. Des interventions alternatives pourraient être considérées dans le contexte guyanais dans le cadre de travaux de recherche ou dans celui d'actions incluses dans le programme d’élimination. Le traitement basé sur un test sérologique pourrait être une option. Ces tests sérologiques permettraient d'identifier des infections récentes (<9 mois) et seraient un reflet du portage d'hypnozoïtes en contexte de faible transmission [46]. L'association à un test diagnostique tel que la PCR serait probablement nécessaire pour ne pas méconnaître une forme aiguë [91].

### Interventions innovantes pour les patients difficiles à atteindre

**Figure 11:**
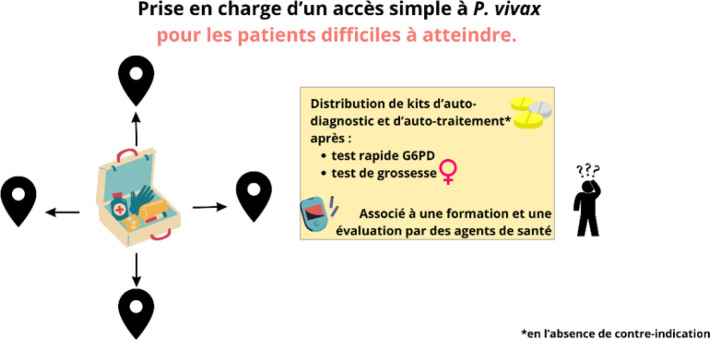
Propositions de prise en charge d'un accès simple à *P. vivax* en zone d'endémie, pour les patients difficiles à atteindre G6PD : Glucose-6-phosphate déshydrogénase

Les stratégies classiques de contrôle du paludisme ne peuvent être mises en œuvre directement auprès de la population travaillant sur les sites d'orpaillage clandestin en raison de contraintes logistiques (éloignement des sites jusqu’à cinq jours de pirogue et/ou de marche), règlementaires (seul un professionnel de santé peut réaliser un test diagnostique et il en manque sur le territoire), sécuritaires, etc. [26]. Un projet innovant a évalué une stratégie renversant le dogme soignant-soigné, basée sur la distribution de kits d'auto-diagnostic et d'auto-traitement du paludisme. Les kits étaient distribués aux orpailleurs après une formation à leur bonne utilisation par des médiateurs sur les lieux de passage transfrontaliers, et non pas directement sur les sites d'orpaillage. Ce projet appelé Malakit a permis la distribution de plus de 4 700 kits à plus de 3 700 personnes entre 2018 et 2020. Le recours au test diagnostique avec un traitement par ACT a augmenté de 54,2 % à 68,1 % (p = 0,011) et le kit était correctement utilisé dans 71,7 % (65,8-77,7) des cas [23]. Ce projet a permis d'accélérer la diminution de l'incidence et de la prévalence du paludisme dans cette population, notamment concernant l'espèce *P. falciparum* [47]. Pour lutter contre *P. vivax* à présent prédominant, cette approche a été adaptée et mise en œuvre depuis 2023. Le projet Curema consiste aujourd'hui à distribuer des kits contenant un traitement présomptif anti-reviviscence aux personnes potentiellement porteuses d'hypnozoïtes [77]. Après un test rapide G6PD et un test de grossesse chez les femmes, les personnes incluses reçoivent primaquine ou tafénoquine avec un suivi des évènements indésirables (Fig. 11). Ces projets innovants sont d'intérêt pour d'autres régions du monde avec des problématiques similaires. Ils sont appelés à être exportés vers d'autres régions endémiques tels que l’Inde, le Sénégal ou le Brésil. Dans notre région, la pérennisation de ces actions en tant qu'intervention de santé publique sous l’égide de l’ARS est en cours, en partenariat avec les CDPS et les associations impliquées dans la médiation et ce, en dehors du cadre de la recherche.

### La lutte antivectorielle (LAV)

Chaque diagnostic de paludisme conduit à une notification auprès du service de la Direction de la démoustication et des actions sanitaires (DDAS) de la Collectivité territoriale de Guyane (CTG). Celle-ci a pour missions la surveillance entomologique et la LAV.

La surveillance entomologique est continue *via* des prélèvements périodiques des gîtes larvaires pour *An. darlingi.* (Fig. 1). Ces gîtes peuvent être variés : bassins, canaux, criques, savanes inondées, marécages, berges de cours d'eau, etc. En cas de détection de larves au sein des gîtes, un traitement par le *Bacillus thuringiensis israelensis (BTI)* – bactérie utilisée comme agent de lutte biologique – est réalisé dans le but de réduire la densité vectorielle.

La LAV, quant à elle, comprend essentiellement l'association des moustiquaires imprégnées d'insecticide (gérées par l’ARS) et la pulvérisation d'insecticide à effet rémanent (à base de pyréthrinoïde) intra et/ou extra-domiciliaire [95]. La LAV est déclenchée :

- immédiatement en cas de signalement d'un cas de paludisme à proximité d'un gîte larvaire déjà sous surveillance active;
- ou après enquête épidémiologique par la DDAS sur le lieu présumé de contamination lors d'un signalement d'un cas à distance d'un gîte larvaire connu. Le délai médian entre le diagnostic de paludisme et la démoustication est de 14 jours et comprend un délai de 7 jours pour les notifications des cas à la CTG, puis de 6 jours supplémentaires pour la réalisation de l'enquête épidémiologique.

Les pulvérisations à base de pyréthrinoïde sont réalisées au domicile du patient et aux alentours : le périmètre dépend de la zone (urbaine ou périurbaine) et de l'enquête épidémiologique. De plus, des pulvérisations préventives peuvent être effectuées dans les zones de présence de vecteurs mais sans contamination encore déclarée, notamment à proximité des voies de navigation utilisées par les orpailleurs. A ce jour, aucune résistance des vecteurs aux insecticides chimiques n'a été identifiée en Guyane [93].

L'objectif d’élimination nécessite une optimisation de la LAV par une plus large distribution des moustiquaires et une évolution vers une notification quotidienne des cas pour réduire le délai avant démoustication. D'autres actions comme le drainage d’étendues d'eaux stagnantes et les pulvérisations préventives sont envisagées. (Fig. 9).

## Conclusion

Face à la recrudescence du paludisme à *P. vivax* qui sévit en Guyane depuis fin 2023, le chemin vers l’élimination du paludisme s'annonce semé d'embûches. Cependant, d'importantes avancées ont été réalisées en peu de temps, incluant la sécurisation de l'approvisionnement en chloroquine, artéméther/luméfantrine et primaquine, l'allègement partiel des contraintes réglementaires dans le suivi administratif du traitement par primaquine, la mise en œuvre du dosage de l'activité de la G6PD dès la phase initiale et le déploiement de stratégies de dépistage actif. Par ailleurs, plusieurs actions clés sont en cours de mise en place, telles que l>obtention de l>autorisation de mise sur le marché pour la primaquine pour remplacer la contraignante AAP, la réalisation des dosages enzymatiques G6PD directement en Guyane *(versus* en France hexagonale), l'introduction de tests de dosage rapide de l'activité G6PD, la distribution de kits d'auto-diagnostic et d'autotraitement pour les patients difficiles à atteindre, ainsi que le renforcement de l'approche communautaire et de la médiation en santé.

Les politiques de lutte contre le paludisme en Guyane doivent permettre de déployer des stratégies adaptées aux réalités locales : sociales, environnementales, géographiques et démographiques. Le développement d'une équipe opérationnelle et réactive conséquente, incluant des professionnels médicaux, paramédicaux et des médiateurs en santé, dotée de protocoles adaptés aux niveaux épidémiques, est crucial pour circonscrire de manière réactive et efficace les foyers de transmission. La marche vers l’élimination du paludisme en Guyane est encore longue mais faisable grâce à de l'adaptabilité, de l'innovation et aux équipes engagées sur le territoire.

## Remerciements

Nous remercions l'ensemble de l’équipe de maladies infectieuses notamment Frédégonde About pour ses conseils, le laboratoire du Centre hospitalier de Cayenne (et spécifiquement Stéphanie Weber pour ses remarques) et Stanislas Talaga de l’Institut Pasteur pour son expertise concernant *Anopheles darlingi.* Nous remercions également pour leurs relectures avisées : Élodie Chane-Ki de la pharmacie hospitalière du CHC, Louise Hureau-Mutricy de l’EMSPEC et Johana Restrepo de la CTG.

## Sources de financement

Aucune

## Contribution des auteurs et autrices

Conception texte : LD, LE Conception figures : LD

Rédaction : LD, LM, LE, HT, AS, AD, YLS, VC, BB, MB, BD, MO, JR, EM, MD Révision : LD, LM, LE, HT, AS, AD, YLS, VC, YA, BB, MB, BD, MO, JR, EM, MD, RNK, LC, OL, PLT, PA, FM, JBD, FD, DP

## Déclaration de liens d'intérêts

Les auteurs déclarent ne pas avoir de liens d'intérêt.
