# Supplementary material for: Malaria control in French Guiana: What are the challenges in this last endemic French territory in 2024?
Source: Med Trop Sante Int. 2025 Mar 20;5(1):mtsi.v5i1.2025.536. [Article in French] doi: 10.48327/mtsi.v5i1.2025.536 (PMC12001996; doi:10.48327/mtsi.v5i1.2025.536)
Supplement: Related texts

# Research article: Malaria control in French Guiana: What are the challenges in this last endemic French territory in 2024?

**Table:** Abbreviation list

CAA	Compassionate access authorization
EAA	Early access authorization
ACT	Artemisinin-based combination therapy
AMM	Marketing authorization for a medicinal product
ANSM	French National Agency for the Safety of Medicines and Health Products
ARS	Institution responsible for the regional management of the health system
ATU	Use of an unapproved drug or medical device under special forms of investigational new drug applications (IND) or IDE application for devices, outside of a clinical trial)
CDPS	Off-site prevention and care center
CHC	Cayenne Hospital
CHK	Kourou Hospital
CHOG	West Guiana Hospital Centre
CNR	National Reference Centre
CORRUSS	Operations center for receiving and regulating responses to health and social emergencies
CRF	French Red Cross
CTG	Collectivité territoriale de Guyane
CQ	Chloroquine
DDAS	Direction de la démoustication et des actions sanitaires
DGAS	Directorate General for Civil Aviation
DGS	General directorate for health
EMSPEC	Équipe mobile de santé publique en commune
FAG	Forces armées en Guyane
FMGE	Thin and Thick Blood Films
G6PD	Glucose-6-phosphate déshydrogénase
HAS	French National Authority for Health
HCSP	French High Council on Public Health
IDE	State-registered nurse
IPG	Institut Pasteur de Guyane
IV	Intravenous
VC	Vector control
WHO	World Health Organization
PCR	Polymerase chain reaction
PMQ	Primaquine
PUI	Internal-use pharmacy
PUT-RD	Therapeutic use and data collection protocol
SAU	Emergency department
SpF	Santé publique France
SPILF	Société de pathologie infectieuse de langue française
RDT	Rapid diagnostic test
TFN	Tafenoquine
RODT	Rapid orientation diagnostic test

## Introduction

French Guiana, an ultramarine territory of France and an outermost region of the European Union, is located in northeastern South America at an equatorial latitude. It is bordered by rivers to the southeast by Brazil (Oyapock River) and to the northwest by Suriname (Maroni River) (Fig. 1). This area, part of the Guiana Plateau, remains the last bastion of endemic malaria transmission in France [29]. However, while Mayotte has had no autochthonous malaria transmission since 2020 [51], the active circulation of *Plasmodium vivax* in French Guiana has accelerated since the end of 2023 [80].

**Figure 1:** Health network in French Guiana and main malaria transmission zones in 2023-2024

The geographical, economic and social situation of this territory complicates the elimination of malaria, which France has committed to by 2025, according to WHO guidelines [98, 99]. Its equatorial climate and extensive Amazon rainforest cover –more than 95%– mean that the human and natural environments are largely intertwined, including the urbanized coastline [30]. The main malaria mosquito in the region, *Anopheles darlingi*, is found in peri-forest zones, degraded forest and savannahs. It is responsible for bites between dusk and dawn, but recent descriptions report diurnal bites with this mosquito [27, 72]. Other potentially vector-carrying *Anopheles* species have also been described, such as *An. nuneztovari, An. oswaldoi* or *An. aquasalis* [12, 28].

The proximity between forest dwellers and vectors (especially *An. darlingi)* is constant. Thus, between the late 2010^s^ and the early 2020^s^, malaria transmission in French Guiana occurred mainly in two types of contexts (Fig. 1). First, it was associated with gold panning activities in forested areas, where gendarmes and the French Guiana Armed Forces (FAG) are also deployed to control these (often illegal) gold mining activities [22, 25, 56, 71, 75]. Indirectly, this transmission may also extend to localities where the logistical backbone for gold panning is located. Secondly, malaria transmission affected populations living along the Oyapock and Maroni rivers, with significant transboundary movement and frequent activity in peri-forest areas [33, 62, 64, 65].

French Guiana is also a place of ethnic and cultural diversity, thanks to its mixed history and successive regional and international migrations [15, 29, 85]. Recent decades have been marked by a demographic explosion (the total population has doubled in 25 years, from 150,000 in 1997 to about 300,000 in 2023) [41], which has exacerbated social inequalities. More than half of the population lives below the poverty line, and 29% live in extreme poverty [42]. Most of the population is concentrated on the coast and along the border rivers. About 60,000 people live in inland communities, mainly along rivers [41, 85]. Health care is provided primarily by three coastal hospitals: Centre hospitalier de Cayenne (CHC), Centre hospitalier de l'ouest guyanais (CHOG in Saint Laurent du Maroni) and Centre hospitalier de Kourou (CHK). The health network in the interior is made up of three local hospitals (Maripasoula, Grand-Santi and Saint-Georges de l’Oyapock) and fourteen Centres délocalisés de prévention et de soins (CDPS), most of which are linked to the coast by river or air and are coordinated by the CHC (Fig. 1).

The aim of this article is to put into perspective the application of the French recommendations in this area and to describe the innovative interventions implemented in a unique geographical, environmental and social context.

## Epidemiology

### Malaria in French Guiana

In recent decades, French Guiana has seen a gradual decline in the number of malaria cases, from more than 4,000 cases per year in the 2000s to 51 cases in 2022 (incidence nadir of 0.18 % reached in 2022) (Fig. 2) [29, 81, 82]. The trend was reversed in the last quarter of 2023, with an increase in incidence not seen since 2018 (annual incidence 2023: 1.2‰) [79, 80]. Historically, *Plasmodium falciparum* was the predominant species until the mid-2000s. Currently, *P. vivax* is the predominant species (>93%) [64, 79] (Fig. 2). A few cases of *P. malariae* have been described more anecdotally and are generally transmitted in the open forest in association with a selvatic cycle [94]. It is important to emphasize that the number of cases reported by surveillance data does not fully reflect the actual cases of malaria in French Guiana, particularly those diagnosed on both sides of the border rivers, as well as cases reported in Europe on return from French Guiana (travelers, military personnel, etc.) [47, 92].

**Figure 2:** Number of malaria cases in French Guiana, 1999-2024. Sources used: [69, 83, 84, 6, 43]. *P. falciparum* / *Non-falciparum* malaria species distribution data not available before 2005

Recurrence of *P. vivax* can be explained by several phenomena: treatment failure, reinfection or reviviscence (reactivation of hepatic hypno-zoites) [1]. Reviviscence is defined as an attack of *P. vivax* that occurs three weeks to one year after the previous attack. In French Guiana, the majority of relapses occurred within the first three months after the initial attack (95% in 20-23), consistent with what has been observed on the South American continent [9, 18, 37, 65]. In the epidemic that occurred between January and June 2024, relapses accounted for 40% of cases (127 cases out of a total of 319) [83].

### Malaria linked to gold panning

Illegal gold miners are a population particularly affected by malaria in French Guiana. Nevertheless, assessment of the epidemiological situation on gold panning sites remains approximate, as surveillance data do not reflect the actual epidemiological situation. Indeed, a large proportion of this population resort to self-medication when they feel ill, or go to health centers in Suriname or Brazil for consultations [25]. Since the implementation of the Malakit project (2018-2020), which involved the distribution of malaria self-diagnosis and self-treatment kits to illegal gold miners, some of them are also diagnosing and treating themselves without notification in the surveillance system [23]. Several descriptive cross-sectional studies (Orpal) were carried out in 2015, 2018, 2019 and 2022 at gold panners’ crossing points on the two border rivers. The results, which reflect the situation at certain gold panning sites, showed a PCR-positive prevalence of 22.3% in 2015 versus 5.3% in 2019 for the Guyana-Suriname border, and 3.9% in 2018 versus 2.5% in 2019 for the Brazil-Guyana border. Data for 2022 are pending. prior to the Malakit project (2018-2020), *P. falciparum* was most prevalent (58%) at gold-mining sites while *P. vivax* is now predominant (85%) [23, 25, 64].

### French Guiana Armed Forces (FAG), sentinels of malaria

The FAG play a veritable sentinel role in the transmission of malaria in the gold-bearing zone. Since 2008, military personnel have been involved in operations aimed at reducing illegal gold mining in French Guiana by disrupting the logistics of gold mining operations in primary forests through the Anaconda and Harpie operations. To achieve this, 400 military personnel are permanently deployed in the area, exposed to infected vectors due to their immersion in the deep forest and proximity to gold-digging populations, known to be a reservoir of malaria. This requires a constant strategy of malaria control in the armed forces through personal antivector protection, chemoprophylaxis with doxycycline, and pre-positioned diagnosis and treatment in the forest [21, 25, 73]. As described below, the FAGs have once again played a sentinel role in the malaria epidemic that French Guiana has been experiencing since the end of 2023.

### A Guyanese *P. falciparum* cluster in June 2023

Although *P. falciparum* is under-represented and infections in recent years have been associated with returns from Africa, French Guiana was alarmed by an outbreak of autochthonous *P. falciparum* transmission in June 2023. This outbreak occurred in a peri-urban area of the commune of Matoury, where *Anopheles* populations are dense. The six patients had not traveled, and four of them had at least one clinico-biological factor of severity. A total of 2/6 patients required transfer to the intensive care unit. No deaths were reported, and 2/6 patients relapsed 20 and 26 days after treatment. Survival and *in vitro* chemosensitivity tests revealed no parasite resistance to artemisinins or lumefantrine. One patient had diarrhea at the time of treatment. Only ongoing plasma studies will be able to confirm proper absorption of the drugs. It should be noted that these cases occurred in an area just a few hundred meters from the Félix Eboué International Airport, but analysis of the resistance profile confirms the Amazonian origin and clonal transmission. With the help of health mediators, active screening using ultrasensitive PCR was offered to 200 residents living within a radius of the cases. None of the PCRs were positive, and the outbreak died out with patient care and reactive vector control (unpublished data). This scenario underscores the risk of resurgence of *P. falciparum* malaria and highlights the importance of an enhanced surveillance system and reactive management, both by mobile malaria teams and vector control teams.

### An epidemic of *P. vivax* in French Guiana since October 2023

Since October 2023, French Guiana has experienced a significant increase in the number of malaria cases, 95% of which are associated with *P. vivax* (Fig. 2). The 5% of cases not associated with *P. vivax* are imported malaria. The first outbreak of the 2023 epidemic detected by the surveillance system was among the FAG operational teams. Despite the FAG's malaria control strategy, 19% of the 378 malaria attacks that occurred between October 2023 and January 2024 involved military personnel [80]. Two large clusters totaled 40 cases of *P. vivax* malaria in deep forest operational areas, with an attack rate of 89% (8/9) for the first cluster and 79% (32/44) for the second cluster. This situation led to a reminder and reinforcement of preventive measures: setting up bivouacs away from high-risk sites, use of long-lasting insecticide-treated ham-mocks-mosquito nets, personal hygiene before nightfall to avoid exposure to bites, wearing long clothes after dark, use of skin repellents, strict adherence to antimalarial chemoprophylaxis. In the rest of the population, cases of *P. vivax* have been reported in areas that had been free of the disease for several years, on the coast and in certain peri-urban areas of the communes of Kourou, Montsinery-Tonnegrande and Roura [78, 80]. The concurrent massive epidemic of dengue fever may also have led to delays in malaria diagnosis due to an overburdened health system and barriers to consultation, with patients reluctant to seek medical advice [8]. In addition, there was a temporary shortage of artemisinin derivatives, a halt in the marketing of chloroquine, and limited access to radical treatment (Box 1).

Box 1 Specific issues concerning access to radical treatment in French Guiana

Since February 2022, the HAS has issued an early access authorization (EAA) for primaquine, which does not have a marketing authorization, after the 2020 cohort ATU. This EAA is subject to compliance with a therapeutic use and data collection protocol (PUT-RD), which includes computerized administrative procedures for each prescription. Upon receipt of the G6PD activity, the prescriber submits the EAA request on a web platform, which requires a number of clinical and biological details. Each EAA request from a physician must be validated by the hospital pharmacist before primaquine can be dispensed. Drug orders involve iterative air deliveries with incompressible logistics time (export procedures, customs, transportation, etc.). for example, the deliveries of primaquine, were delayed by more than a month during the holiday season at the end of 2023 due to the prioritization of food deliveries. During the follow-up period, the prescriber must continue to follow the regulatory procedures and complete the clinico-biological data at D0, D7, D14 and a definitive treatment discontinuation form. Failure by prescribers to complete these forms has led to sanctions by the pharmaceutical company, drastically reducing primaquine supplies and delaying the introduction of radical treatment. At the instigation of the ARS, negotiations between the ANSM and the pharmaceutical laboratory led to a relaxation of the primaquine prescription procedures: the D7 and D14 forms no longer had to be completed and the sanctions were lifted.

Other solutions have been implemented in early 2024 to facilitate overall management:

1) The CHC laboratory has validated the implementation of the enzymatic reference technique for the determination of G6PD activity in the Cayenne laboratory in April 2024, which could provide same-day G6PD assay results.
2) The CNR paludisme of the Institut Pasteur de la Guyane has carried out a study to evaluate the performance of a rapid quantitative G6PD activity test (“STANDARD G6PD”) for the detection of G6PD-deficient patients in comparison with the enzymatic reference method. The device is simple, fast and efficient. The sensitivity/specificity values of the rapid test for detecting deficient males and females are 100% [89-100]/83% [73-93] and 100% [86-100]/90% [83-97], respectively (Lazrek *et al.* unpublished data). The use of these capillary tests could allow the initiation of radical treatment at D0, in combination with chloroquine or ACT, even in remote areas, following the Brazilian recommendations [50] (Fig. 6 and Fig. 8). However, the use of this rapid diagnostic method will require indepth and continuous training for all those involved in diagnosis, given the precautions to be taken to achieve this level of performance.
3) At the instigation of the ARS, the transport route for medicines to French Guiana was secured in order to prioritize pharmaceutical orders. This collaboration, involving the Direction générale de la santé (DGS), the Centre opérationnel de régulation et de réponses aux urgences sanitaires et sociales (CORRUSS) and the Direction générale de l'aviation civile (DGAS), helps to minimize delays in the delivery of medicines.

Several levers remain to be pulled that could have a significant impact on public health.

- The AMM for primaquine is expected to be effective in the coming months, which would drastically reduce the time to radical treatment (as early as D0) and reduce time-consuming administrative procedures for clinicians and pharmacists.
- Measuring G6PD activity as soon as malaria is diagnosed in the emergency department could reduce the time to initiation of radical treatment.
- Shortening the duration of primaquine treatment from 14 to 7 days, which has been implemented in many malaria-endemic countries (but not retained in the 2018 HCSP opinion), could be discussed to promote compliance [36, 50].
- The availability of single-dose tafenoquine -8-ami-noquinoline- could also represent a major benefit in the care pathway by minimizing follow-up time and limiting the number of patients lost to follow-up before radical treatment [9, 10, 16]. Its marketing in France does not appear to be on the agenda at present.
- Finally, in malaria-endemic areas where *P. vivax* is endemic, a systematic G6PD assay could be performed as part of a medical examination for all people living in high-risk areas and recorded in their medical records to facilitate the implementation of curative treatment in the event of a malaria attack (Fig. 8).

### Regional context since 2023: recent developments on the Guiana Plateau

The epidemiological evolution observed in French Guiana between 2023 and the first quarter of 2024 must be interpreted in a regional and continental context. In 2023, a major epidemic broke out in the Brazilian border state of Amapá, particularly affecting the municipalities of Calçoene (907 cases in 2022 vs. 2,637 in 2023) and Oiapoque, the border town with French Guiana (454 cases in 2022 vs. 892 in 2023) [58]. The epidemic had its epicenter in the district of Lourenço (Calçoene), a historical area of legal and informal gold mining [50] (Fig. 1). It was characterized by a preponderance of *P. vivax*, which accounted for 95% of reported cases [93]. In contrast, Suriname had a stable year, with no indigenous transmission in the country since 2021 [67].

On an Amazonian or even continental scale, two other phenomena may have influenced the epidemiologic dynamics of malaria in French Guiana. On the one hand, there were population movements, difficult to quantify, from illegal gold-mining areas in the Yanomami Indian reserve in the Brazilian state of Roraima, which was the subject of major military operations in early 2003 as part of President Lula's policy to strengthen the protection of indigenous populations [49, 48]. These movements are thought to have contributed to the dispersal of a population heavily affected by *P. vivax* to other gold-mining basins in Amazonia, including Amapá and French Guiana [20, 54]. On the other hand, the El Niño climatic phenomenon affected the entire American continent, resulting in an exceptionally dry and hot year in Amazonia [55, 98], which led to the formation of water reservoirs, creating an environment conducive to the reproduction of vectors. We could also mention the impact of the end of intervention studies in gold mining areas, such as the end of Malakit project in 2020 (distribution of malaria self-diagnosis and self-treatment kits to illegal gold miners [see below]) [23]. Thus, the origin of the current *P. vivax* epidemic in French Guiana is likely multifactorial and warrants further investigation.

### Managing elimination and the risk of reintroduction

With the decline in malaria cases between 2000 and 2022, French Guiana has entered the malaria elimination phase (i.e., no indigenous malaria transmission for three consecutive years). In contrast to a control program, the goal of elimination is to interrupt transmission and then prevent reintroduction [59, 96]. The pathway to WHO certification includes three main phases: 1) screening and reactive treatment of cases, co-exposed persons and their environment, 2) detection and targeted response of populations considered to be reservoirs, and 3) prevention of the risk of introduction or reintroduction by screening persons from endemic areas at points of entry into the territory [89, 96, 99]. The border areas of French Guiana remain highly vulnerable to malaria, particularly due to the high mobility of the population and the global regional endemic context that perpetuates possible transmissions [2, 17, 26, 60, 62, 76, 89]. The WHO goal of eliminating malaria in French Guiana by 2025 is in jeopardy because of epidemic phenomena in 2023-2024.

## Clinics

*Plasmodium vivax* infections are generally described as less severe than *P.falciparum* infections. However, the incidence of severe cases varies according to geographic area and sensitivity to antimalarial drug, ranging from 0.6% to 21% in the literature [36, 45, 63, 70, 88]. In French Guiana, the proportion of severe *P. vivax* infections has been estimated at 4% between 2018 and 2020 [18]. The current epidemic seems to have a slightly different profile.

Of the 371 cases of *P. vivax* malaria identified between October 2023 and January 2024, 17% (62/371) were hospitalized in one of the three main hospitals in Guyana. Of the 34 patients admitted to CHC, 16 (47%) presented with at least one WHO severity factor. The most common were jaundice (7), respiratory distress (6), and circulatory failure (4). Of the 34 patients hospitalized, 5 required transfer to the intensive care unit (15%), 8 were treated with intravenous artesunate; no deaths occurred [19]. Further studies will be needed to better describe these unexpected aspects of severity across the territory.

## Parasitologic Diagnosis

### Diagnostic methods

Every patient presenting with fever in French Guiana should be systematically tested for malaria. The diagnosis is made in the laboratory and the result must be available within two hours of receipt of the sample. This is based on the French recommendations to combine a sensitive technique (thick smear or molecular biology) with a thin smear or, failing that, a rapid diagnostic test (RDT) combined with a thin smear [86]. The RDT mainly used in French Guiana is the Sd Bioline Pf/Pan (HRP2/pLDH) antigen. Its sensitivity for diagnosing *P. falciparum* is very good, especially since no HRP2 deletion has been found on the Guyana Plateau [10]. The sensitivity for the diagnosis of *P. vivax* is reported by the WHO to be 91.7%, requiring the malaria test to be repeated on a new sample in cases of high clinical suspicion [87, 97]. In local hospitals or CDPSs, on-site RDT allows treatment to be initiated on a positive result without waiting for microscopy. Each positive case must then be sent to the CHC laboratory for microscopic confirmation and determination of parasitaemia, and then referred to the CNR Malaria.

### Off-site use of malaria RDTs

In areas far from health facilities, diagnostic confirmation becomes more complex. RDTs fall within the scope of off-site biology and should therefore be confirmed by a biologist. However, the derogation decree specific to French Guiana of December 1, 2016 (revised in April 2024) has made it possible to simplify management by giving the malaria RDT the status of a rapid diagnostic orientation test (TROD). Thus, after specific training and accreditation, IDEs and staff of health or prevention structures can carry out malaria antigen detection tests in locations far from any medical biology laboratory in French Guiana [57].

### Active screening with ultrasensitive PCR

Ultrasensitive PCR is used for active screening *(Reactive Test and Treat)* of asymptomatic patients in specific epidemic situations. This involves performing PCR on everyone living in an area identified as “at risk” (by peer assessment) and treating positive individuals (Fig. 3). This active screening is coordinated by the ARS and the PCR is performed by the CNR at the IPG. The most recent screening took place in February-March 2024 in the Amerindian village of Favard (part of the municipality of Roura). Of the 138 inhabitants screened, 11 patients were symptomatic and RDT positive (8%). Four asymptomatic patients were detected by PCR and were referred for treatment.

**Figure 3:** Active screening strategy implemented in French Guiana TDR: Rapid Detection Test; TTBF: Thin and thick blood films; PCR: Polymerase Chain Reaction; ACT: Artemisinin-based Combination Therapy; CQ: chloroquine; G6PD: Glucose-6-phosphate dehydrogenase; PMQ: primaquine; D: day * in the absence of contraindication ** adapted to context and geographical remoteness

The ultimate goal is to immediately treat all cases diagnosed during active screening in order to reduce malaria transmission as quickly as possible.

## Treatment

### Treatment of *P. falciparum* attacks

Treatment of *P. falciparum* malaria is based on national and international recommendations, with artemether-lumefantrine (20 mg/120 mg, 4 tablets at H0, H8, H24, H36, H48, H60 in adults) in the absence of severity criteria, or artesunate IV if severity criteria are present [30] (Fig. 4 and Fig. 5). As recommended by the HCSP in view of the goal of malaria elimination, patients with *P. falciparum* should receive a single dose of primaquine (0.25 mg/kg) for “altruistic” purposes, *i.e.* its anti-gametocytic effect in reducing parasite transmission to *Anopheles* vectors [5, 38]. The arsenimol-piperaquine combination, widely used in France, was used in FAG in the late 2010s. Its use was suspended following the identification of numerous treatment failures related to the non-negligible proportion of piperaquine-resistant *P.falciparum* on the Guyana Plateau [32]. Although not widely used in the health care system, piperaquine resistance may have been selected through massive self-medication by gold miners on the Guyana Plateau since the early 2000^s^ [24].

**Figure 4:** Suggestions for the management of simple access to *P. falciparum* in endemic areas TTBF: Thin and thick blood films; PMQ: primaquine; D: day

**Figure 5:** Suggested management of severe *P. falciparum* infection in endemic areas TTBF: Thin and thick blood films; ACT: Artemisinin-based Combination Therapy; G6PD: Glucose-6-phosphate dehydrogenase; ATU: Temporary Authorization for Use; PMQ: primaquine; SDG: Signs of seriousness; PSE: electric syringe pump; D: day

### Treatment of *P. vivax* attacks

Until recently, *P. vivax* malaria attacks were treated with chloroquine at a dose of 25 mg/kg over 3 days (D1: 10 mg/kg/d - D2: 10 mg/kg/d - D3: 5 mg/kg/d). The end of its commercialization in 2022 has necessitated changes in protocols. For example, artemether-lumefantrine (20 mg/120 mg) is currently used at the same dose as for *P. falciparum* attacks [29, 30] (Fig. 6 and Fig. 7). This paradigm shift appears to have implications for compliance and treatment failure. As a reminder, treatment failure is currently defined by Santé publique France as a second attack within three weeks of the previous attack [79].

**Figure 6:** Suggested management of simple access to P. *vivax* in endemic areas, according to access to the healthcare system TDR: Rapid Detection Test; TTBF: Thin and Thick Blood Films; ACT: Artemisinin-based Combination Therapy; CQ: chloroquine; G6PD: Glucose-6-phosphate dehydrogenase; PMQ: primaquine; D: day

**Figure 7:** Suggested management of severe *P. vivax* infection in endemic areas TTBF: thin and thick blood films; ACT: Artemisinin-based Combination Therapy; G6PD: Glucose-6-phosphate dehydrogenase; ATU: Temporary Authorization for Use; PMQ: primaquine; SDG: Signs of seriousness, PSE: electric syringe pump

Of 59 soldiers treated with ACT, 61% (36/59) had a second attack, mostly within the first month, although they would not have been re-exposed during this period (19% treatment failure, 81% relapse). Relapses occur early: a median of 27 days in infectious disease patients compared to a median of 63 days in the same population treated with chloroquine in 2018-2020 [18]. Also of note are several relapses after well-managed treatment in patients initially treated with ACT (unpublished data).

In response to the current health crisis, and following the discontinuation of chloroquine marketing, Guyana's ARS has obtained its availability under compassionate access authorization (CAA) status. Chloroquine is an important molecule because of its efficacy against *P. vivax* attacks, its potential to reduce the risk of treatment failure and revival, and its synergistic effect with primaquine [3, 16, 35, 46, 74]. It is therefore available from the hospital pharmacy after completion of the regulatory forms. Chloroquine urgently needs to be relicensed so that it can be made available nationwide (including in outpatient clinics and remote areas) and administered without delay.

### Radical treatment

*Plasmodium vivax* produces hepatic hypnozoites that can reappear a few weeks to a few months after treatment with a schizonticide. To effectively eradicate them and prevent relapses, radical treatment with an 8-aminoquinoline is used, of which only primaquine is available in France. Primaquine can cause hemolytic anemia in severe glucose-6-phosphate dehydrogenase (G6PD) deficiency. G6PD activity testing is therefore recommended by the WHO and required by the French National Agency for the Safety of Medicines and Health Products (ANSM) before prescribing primaquine. The French High Council of Public Health (HCSP) recommends measuring G6PD activity on day 14 of the malaria attack to avoid initial hemolysis, which could overestimate G6PD levels. However, radical treatment follow-up in French Guiana faces a large number of patients who are lost to follow-up, leading to repeated relapses that can perpetuate transmission chains. To illustrate, of 113 malaria attacks studied at CHC between 2018 and 2020, the median time between treatment with chloroquine or ACT and primaquine was 28 days. Of the 113 malaria attacks, 30 patients (27%) were lost to follow-up before primaquine treatment, and 40 patients (3%) experienced at least one relapse [18].

In this context, the possibility of performing early G6PD assays (as soon as malaria was diagnosed) was investigated. This retrospective study found no variations in G6PD activity between the start of treatment and D28: no patient classified as G6PD deficient at baseline was found to be deficient at D14. On the contrary, it highlighted cases of false positives: patients falsely classified as G6PD deficient at baseline, who eventually normalized during follow-up. This would suggest the possibility of measuring G6PD activity early in the course of a *P. vivax* attack and rechecking the assay in case of initial deficiency [18].

At the onset of the 2023 malaria epidemic in French Guiana, administrative obligations and logistical difficulties (Box 1) added to the already existing surveillance difficulties and significantly delayed the implementation of radical treatment. In this context, and in the light of recent reassuring data in the literature [13, 18, 52], early G6PD testing with detailed patient information and close clinical-biological follow-up was introduced (Fig. 6). Several other levers for diagnostic and therapeutic optimization are still pending and could have a significant impact on public health (Box 1, Fig. 8, Fig. 9). In French Guiana, as in South America, the prevalence of G6PD deficiency is low, <2% [11, 18]. The G6PD assay is expressed in units of enzyme per gram of hemoglobin (U/g Hb). G6PD activity is interpreted as a percentage. Therefore, the clinician must derive the percentage activity according to the standard of each laboratory. For example, according to the standards currently used by the Biomnis laboratory processing samples in French Guiana, 100% G6PD activity corresponds to the laboratory's cut-off value of 10 U/g Hb (i.e. 8 U/g Hb - 80%, 3 U/g Hb - 30%, 1 U/g Hb - 10%). The primaquine dosage schedule according to G6PD activity and HCSP recommendations is shown in Figure 10.

**Figure 8:** Proposed diagnostic and therapeutic regimens for the management of *P. vivax* infections in French Guiana TTBF: Thin and Thick Blood Films; ACT: artemisinin-based Combination Therapy; CQ: chloroquine; G6PD: Glucose-6-phosphate dehydrogenase; PMQ: primaquine; TFN: tafenoquine; TDR: Rapid Detection Test

**Figure 9:** Levers for optimizing malaria management to achieve elimination, considering the challenges in the region

**Figure 10:** Radical treatment of a single *P. vivax* episode in adults based on G6PD activity according to HCSP 2018 recommendations [4, 39, 99] G6PD: Glucose-6-phosphate dehydrogenase; PMQ: primaquine; U: unit; Hb: hemoglobin ** Example: if laboratory G6PD standard = 10 U/g Hb: G6PD 10 U/g Hb = 100% G6PD activity; G6PD 8 U/g Hb = 80% G6PD activity; G6PD 3 U/g Hb = 30% G6PD activity; G6PD 1 U/g Hb = 10% G6PD activity

### Coastal management

Diagnosis and initiation of first-line treatment (ACT or CQ) for *P. vivax* malaria attacks on the coast are often made in emergency departments or outpatient clinics. Patients are referred to the Infectious Diseases Department on D3 for further management: G6PD testing, initiation of primaquine, and regular follow-up until the end of treatment –D14 of primaquine– (Fig. 8). The current organization delays the G6PD assay to D3 and thus the radical treatment. In the case of *P. vivax* infection, a protocol with G6PD dosing already in the emergency department should be discussed in order to optimize radical treatment.

### Management in remote communities

Malaria management in inland communities is complicated by the isolation of the population. Patients may travel several hours or days by pirogue or quad bike to reach the health center, making medical follow-up more complex [14, 85]. In addition, there are difficulties in accessing radical treatment (Box 1), difficulties in contacting patients after treatment (use of foreign phone numbers, insecure phone network…), and logistical challenges. The pirogues or planes that connect the CDPSs to the coast sometimes operate on a weekly basis. Their regularity depends on climatic conditions (river levels, flight conditions) or organizational factors (recent bankruptcy of the intra-Guyanese airline, airline strikes, etc.). Thus, despite the intervention of health mediators tasked with reaching out to patients, all these difficulties lead to multiple missed appointments and very low primaquine coverage [34]. It remains possible that these visual impairments will eventually be treated in Brazil or Suriname due to large cross-border population movements, but this remains uncertain and unquantifiable. CDPSs also suffer from a lack of human resources and high turnover of medical and paramedical teams, which complicates continuity of care [15]. The complexity also lies in the training of these health professionals -non-infectiologists, often from France- who have to understand in record time the specific characteristics of the communities they encounter and the peculiarities of the various epidemics they face.

### The role of chemoprophylaxis

Although it is still mentioned in the HCSP and SPILF recommendations, chemoprophylaxis against malaria for travel to South America is limited with few exceptions [86]. In fact, the risk-benefit ratio of chemoprophylaxis is generally unfavorable in areas with a low risk of *P. falciparum* transmission. Situations that may justify chemoprophylaxis include travel to high-risk areas, isolation in the forest, and special living conditions (e.g., military personnel working in the forest). This information is regularly updated on the website of ARS Guyane, which publishes a malaria risk map for French Guiana coordinated by SpF [7, 29].

## Individual and collective interventions

In an elimination context, it is not enough to simply manage malaria cases identified through passive surveillance (people seeking care for symptoms). Proactive interventions in recent transmission areas are needed to interrupt transmission. In this section, we describe some of Guyana's experiences, as well as some perspectives on interventions that may be relevant in the future for malaria control in the region (Fig. 8).

### Health education and outreach

CHC's mobile community public health teams (EMSPEC) were created in 2019, following the interest observed during the PALUSTOP study (see below) and the malaria epidemic in Saint-Georges de l’Oyapock in 2017. The aim of these teams is to meet the public health needs of people living in the interior of the country, and they are attached to local hospitals and CDPSs [34, 85]. Each team consists of pairs of mediators and nurses, coordinated by a trio of doctors and nurses. Health mediation is placed at the heart of promotion and prevention activities, thanks to community mediators who adapt activities to local needs and cultures. EMSPEC plays a key role in the fight against malaria thanks to its “out-reach” approach and “out-of-home” interventions. As soon as a patient presents with a malaria attack, the teams meet him or her at home, investigate the case, stress the importance of follow-up, distribute insecticide-treated bed nets (provided by ARS) if available, and accompany the patient throughout the course of treatment. They also raise general awareness about malaria and vector control through booths and outreach campaigns, helping to reduce social and territorial inequalities on a variety of issues. In view of the new geographical distribution of cases, and under the impetus of the ARS, four nurse-mediator pairs are to reinforce this approach within the French Red Cross and the CHC in the coastal sector and among gold panners: awareness raising, in-situ RDT screening, referral to the care pathway, distribution of bed nets, etc.

### Screening and treatment of people considered at risk in target populations

In response to the epidemiological situation in Saint-Georges de l’Oyapock in 2017, mass screening of the general population was conducted as part of the PALUSTOP study. This innovative project evaluated a screening and treatment strategy by studying malaria carriage (symptomatic and asymptomatic) by PCR in a cohort of more than 1,200 people followed for more than one year [62]. This study analyzed the value of different diagnostic tools: serology, RDTs and PCR. While RDTs lacked sensitivity in detecting *P. vivax* in active screening (9.9% of 91 PCR-positive cases detected by RDT), their combination with new tools (PCR or serology) seemed relevant in an elimination strategy [60, 91]. While RDT is sufficiently sensitive to detect symptomatic patients, its sensitivity is low in pauci or asymptomatic forms, especially in cases of low parasitaemia [90]. This strategy of mass screening and treatment could be repeated in the current epidemic context. This study made it possible to spatialize the risk of malaria on the outskirts of this cross-border village [62] and to identify specific risk profiles (young Amerindians) or risk factors (logging activities, travel to Brazilian Amerindian areas) [62, 89]. These findings underscored the importance of coordinating cross-border control efforts and prompted the creation of an epidemiologic surveillance system between French Guiana and Brazil [76]. Ethno-pharmacological analyses and a survey of knowledge and practices have revealed major disparities between ethnic groups, implying the need to personalize prevention and care tools through participatory actions and the involvement of community health mediators [33, 61, 66]. Active PCR screening around cases is one of the intervention strategies used by the ARS in response to epidemiological signals or health alerts. This intervention model, widely supported by the WHO, was implemented for the first time outside the research context in 2023 in French Guiana. It requires close collaboration between the ARS, the CRF malaria mobile team (created in April 2024 and composed of a project coordinator, a nurse and mediators), the CHC or CHK doctors, the CHC laboratory, the IPG malaria CNR and health mediators (EMSPEC or volunteers from the DAAC association). This model is currently used on a weekly basis at one of the fallback sites in a goldfield near Kourou, an important transit area for remote populations heavily affected by malaria. In practice, symptomatic and RDT-positive individuals are immediately treated with ACT and primaquine after undergoing G6PD RDT to rule out any deficiency. Diagnosis is confirmed *a posteriori* by swab and thick drop. Active PCR screening is offered to RDT-negative persons living in the vicinity of positive cases or who have been associated with gold panning in the past 12 months, and treatment is organized if positive. Weekly clinical and biological follow-up is carried out on site for all patients receiving primaquine (Fig. 3). This intervention has detected many cases of malaria and needs to be evaluated to organize its sustainability and refine the cost-benefit ratio [31, 96].

However, while the acute phases of malaria attacks are easily detected, no biological test can detect the carriage of dormant forms (hypnozoites). This is the main obstacle to the elimination of *P. vivax.* As a result, recently infected individuals at risk of relapse may escape mass screening by RDT, PCR or microscopy. Mass treatment strategies (targeted or non-targeted) may be relevant for elimination, but they must be weighed against the contraindications of radical treatment [40, 44, 68]. Alternative interventions could be considered in the Guyanese context, either as part of research or as part of interventions included in the elimination program. Treatment based on serologic testing could be an option. These serologic tests could identify recent infections (<9 months) and reflect hypnozoite carriage in a context of low transmission [46]. The combination with a diagnostic test such as PCR would probably be necessary in order not to miss an acute form [91].

### Innovative interventions for hard-to-reach patients

Conventional malaria control strategies cannot be implemented directly with the population working on clandestine gold mining sites due to logistical constraints (sites are up to five days away by pirogue and/or on foot), regulatory constraints (only a health professional can perform a diagnostic test and there is a shortage of them in the area), security constraints, etc. [26]. One innovative project evaluated a strategy that overturned the caregiver-patient dogma based on the distribution of malaria self-diagnosis and self-treatment kits. The kits were distributed to gold miners after training in their proper use by facilitators at border crossings, rather than directly at the gold mining sites. The Malakit project will distribute more than 4,700 kits to more than 3,700 people between 2018 and 2020. Use of the diagnostic test with ACT treatment increased from 54.2% to 68.1% (p = 0.011), and the kit was used correctly in 71.7% (65.8-77.7) of cases [23]. This project has contributed to an accelerated reduction in the incidence and prevalence of malaria in this population, particularly with regard to *P.falciparum* species [47]. This approach has been adapted and implemented since 2023 to combat the now predominant *P. vivax*. Today, the Curema project involves the distribution of kits containing a presumptive antiretroviral treatment to individuals potentially carrying hypnozoites [77]. After a rapid G6PD test and a pregnancy test in women, those enrolled receive primaquine or tafenoquine, with follow-up for adverse events (Fig. 11). These innovative projects are of interest to other regions of the world with similar problems. They are likely to be exported to other endemic regions such as India, Senegal and Brazil. In our region, these initiatives are currently being developed as public health interventions under the auspices of the ARS, in partnership with CDPSs and associations involved in mediation, outside the framework of research.

**Figure 11:** Suggested management of simple access to *P. vivax* in endemic areas, for hard-to-reach patients G6PD: Glucose-6-phosphate dehydrogenase

### Vector control (VC)

Any diagnosis of malaria leads to the notification of the Department of Mosquito Control and Sanitary Action (DDAS) of the Territorial Collectivity of French Guiana (CTG). This department is responsible for entomological surveillance and VC.

Entomological surveillance is continuous, with periodic sampling of *An. darlingi* breeding sites (Fig. 1). These sites can be varied: ponds, canals, streams, flooded savannahs, swamps, river banks, etc. When larvae are detected at breeding sites, they are treated with *Bacillus thuringiensis israelensis* (BTI) –bacteria used as a biological control agent– to reduce vector density.

VC is essentially a combination of insecticide-impregnated bed nets (managed by ARS) and intra-and/or extra-domiciliary residual insecticide spraying (pyrethroid-based) [95]. VC is triggered :

- immediately when a case of malaria is reported in the vicinity of a breeding site already under active surveillance;
- or after an epidemiological investigation by the DDAS at the suspected site of contamination, if a case is reported at a distance from a known larval breeding site. The median time between diagnosis of malaria and mosquito control is 14 days, including 7 days for case reporting to the CTG and a further 6 days for epidemiologic investigation.

Pyrethroid-based spraying is carried out in and around the patient's home: the perimeter depends on the area (urban or peri-urban) and the epidemiological survey. Preventive spraying can also be carried out in areas where vectors are present but contamination has not been reported, particularly near transport routes used by gold miners. To date, no vector resistance to chemical insecticides has been detected in French Guiana [93].

The elimination target requires optimization of VC through wider distribution of bed nets and a shift to daily case reporting to reduce the delay in mosquito control. Other measures such as drainage of stagnant water and preventive spraying are also envisaged. (Fig. 9).

## Conclusion

With the resurgence of *P. vivax* malaria in French Guiana since the end of 2023, the road to malaria elimination is fraught with difficulties. However, significant progress has been made in a short period of time, including securing the supply of chloroquine, artemether/lumefantrine and primaquine, partially easing regulatory constraints on the administrative monitoring of primaquine treatment, implementing G6PD activity testing from the initial phase, and implementing active screening strategies. In addition, several key interventions are underway, including obtaining marketing authorization for primaquine to replace the restrictive EAA, performing G6PD enzyme assays directly in French Guiana (rather than in mainland France), introducing rapid G6PD activity assays, distributing self-diagnosis and self-treatment kits for hard-to-reach patients, and strengthening community outreach and health education.

Malaria control policy in French Guiana must allow the implementation of strategies adapted to local conditions: social, environmental, geographical and demographic. The development of a strong operational and reactive team, including medical and paramedical professionals and health mediators, with protocols adapted to the level of the epidemic, is crucial for the reactive and effective containment of transmission outbreaks. The road to malaria elimination in French Guiana is still long, but it can be traveled thanks to the adaptability, innovation and commitment of local teams.

## Acknowledgements

We would like to thank the Infectious Diseases team, in particular Frédégonde About for her advice, the Cayenne Hospital laboratory (and specifically Stéphanie Weber for her comments) and Stanislas Talaga of the Institut Pasteur for his expertise on *Anopheles darlingi.* We would also like to thank Élodie Chane-Ki of the CHC hospital pharmacy, Louise Hureau-Mutricy of the EMSPEC and Johana Restrepo of the CTG for their expert reviews.

## Funding

None

## Authors’ contributions

Text design: LD, LE Figure design: LD

Editing: LD, LM, LE, HT, AS, AD, YLS, VC, BB, MB, BD, MO, JR, EM, MD

Revision: LD, LM, LE, HT, AS, AD, YLS, VC, YA, BB, MB, BD, MO, JR, EM, MD, RNK, LC, OL, PLT, PA, FM, JBD, FD, DP

## Declaration of interests

The authors declare no conflict of interests.
